# Removal of *N*-linked glycans in cellobiohydrolase Cel7A from *Trichoderma reesei* reveals higher activity and binding affinity on crystalline cellulose

**DOI:** 10.1186/s13068-020-01779-9

**Published:** 2020-08-06

**Authors:** Bartłomiej M. Kołaczkowski, Kay S. Schaller, Trine Holst Sørensen, Günther H. J. Peters, Kenneth Jensen, Kristian B. R. M. Krogh, Peter Westh

**Affiliations:** 1 Roskilde University, INM, Universitetsvej 1, Building 28, 4000 Roskilde, Denmark; 2 Department of Biotechnology and Biomedicine, Technical University of Denmark, Building 224, 2800 Kgs. Lyngby, Denmark; 3 Novozymes A/S, Biologiens Vej 2, 2800 Kgs. Lyngby, Denmark; 4 Department of Chemistry, Technical University of Denmark, Kemitorvet, 2800 Kgs. Lyngby, Denmark

**Keywords:** GH7 cellulase, *Trichoderma reesei* Cel7A, *Aspergillus oryzae*, *N*-Glycosylation, Heterogeneous interfacial enzyme kinetics, MD

## Abstract

**Background:**

Cellobiohydrolase from glycoside hydrolase family 7 is a major component of commercial enzymatic mixtures for lignocellulosic biomass degradation. For many years, *Trichoderma reesei* Cel7A (*Tr*Cel7A) has served as a model to understand structure–function relationships of processive cellobiohydrolases. The architecture of *Tr*Cel7A includes an *N*-glycosylated catalytic domain, which is connected to a carbohydrate-binding module through a flexible, *O*-glycosylated linker. Depending on the fungal expression host, glycosylation can vary not only in glycoforms, but also in site occupancy, leading to a complex pattern of glycans, which can affect the enzyme’s stability and kinetics.

**Results:**

Two expression hosts, *Aspergillus oryzae* and *Trichoderma reesei*, were utilized to successfully express wild-types *Tr*Cel7A (WT_*Ao*_ and WT_*Tr*_) and the triple *N*-glycosylation site deficient mutants *Tr*Cel7A N45Q, N270Q, N384Q (Δ*N*-glyc_*Ao*_ and Δ*N*-glyc_*Tr*_). Also, we expressed single *N*-glycosylation site deficient mutants *Tr*Cel7A (N45Q_*Ao*_, N270Q_*Ao*_, N384Q_*Ao*_). The *Tr*Cel7A enzymes were studied by steady-state kinetics under both substrate- and enzyme-saturating conditions using different cellulosic substrates. The Michaelis constant (*K*_*M*_) was consistently found to be lowered for the variants with reduced *N*-glycosylation content, and for the triple deficient mutants, it was less than half of the WTs’ value on some substrates. The ability of the enzyme to combine productively with sites on the cellulose surface followed a similar pattern on all tested substrates. Thus, site density (number of sites per gram cellulose) was 30–60% higher for the single deficient variants compared to the WT, and about twofold larger for the triple deficient enzyme. Molecular dynamic simulation of the *N*-glycan mutants *Tr*Cel7A revealed higher number of contacts between CD and cellulose crystal upon removal of glycans at position N45 and N384.

**Conclusions:**

The kinetic changes of *Tr*Cel7A imposed by removal of *N*-linked glycans reflected modifications of substrate accessibility. The presence of *N*-glycans with extended structures increased *K*_*M*_ and decreased attack site density of *Tr*Cel7A likely due to steric hindrance effect and distance between the enzyme and the cellulose surface, preventing the enzyme from achieving optimal conformation. This knowledge could be applied to modify enzyme glycosylation to engineer enzyme with higher activity on the insoluble substrates.

## Background

Cellulose-degrading enzymes were first discovered in the secretome of the filamentous ascomycete *Trichoderma reesei* almost 65 years ago [1]. Since then, they have proven essential in commercial enzyme cocktails used in biorefineries that produce fuels and chemicals from lignocellulosic biomass. Due to the complexity, recalcitrance and insolubility of the plant biomass [2], high enzyme titers must be used to ensure efficient biomass hydrolysis, and this challenges the economic feasibility of the process. To overcome this, extensive research has sought to either engineer catalytically more efficient enzymes or to develop more efficient expression hosts such as *Trichoderma reesei* [3], *Saccharomyces cerevisiae* [4] and *Aspergillus niger* [5]. The latter effort has enabled industrial production of cellulases, but usually with a range of isoforms with different apparent molecular weights [6]. This is attributed to the ability of the fungal expression host to decorate proteins with short oligosaccharides. Such post-translational modification called glycosylation can occur at either threonine (T) or serine (S) residues (*O*-glycosylation), or at asparagine (N) residues (*N*-glycosylation), which have a general consensus motif N-X-S/T (where X denotes any amino acid residue except proline) [6].

The dominant cellulase in the secretome of *T. reesei* is the cellobiohydrolase *Tr*Cel7A (EC 3.2.1.176) [7], classified to glycoside hydrolase (GH) family 7 in the CAZy database [8]. The architecture of *Tr*Cel7A includes a catalytic domain (CD), with three *N*-glycosylation sites, which is connected to a carbohydrate-binding module (CBM) through a flexible, heavily *O*-glycosylated linker peptide [9]. Most of the *N*-glycans on *Tr*Cel7A have been characterized as high mannose (Man) type, containing from Man_5–9_ residues linked to a chitobiose core of two *N*-acetylglucosamine (GlcNAc)_2_ units, whereas the *O*-glycans consist mainly of Man_1–4_ randomly distributed in both the linker region and CBM domain with the majority bound to the linker of *Tr*Cel7A [7, 10]. Recently, Amore et al. [3] performed extensive mass spectrometry (MS) characterization of the different *N*-glycoforms of *Tr*Cel7A expressed in *T. reesei*, and this work indicated a broader complexity of *N*-glycans, including the presence of fucose, galactose or additional *N*-acetylglucosamine residues. The glycan complexity is not only influenced by the expression hosts and their extracellular activities of glycosidases and transferases, but also the composition of the growth media [11]. The functional roles of the *N*-glycosylation remain elusive, although it has been shown that disruption of *N*-glycosylation motifs lowers *Tr*Cel7A’s thermal [3, 12] and proteolytic stability [3]. Studies on the influence of *N*-glycosylation on the activity and cellulose adsorption of the CD *Tr*Cel7A have shown either promoting [5, 12] or no effects [3]. Most of these studies [3, 5, 12] evaluated the effect of *N*-glycosylation based on prolonged experiments, usually including the addition of synergistic cellulases. While these conditions mirror technical applications, they might complicate the molecular understanding of *Tr*Cel7A *N*-glycosylation. In the current work, we focused on 1 hour kinetic measurements, which have proven to provide a reasonable proxy for initial rates in comparative kinetic studies of cellobiohydrolases [13]. We performed an extensive biochemical characterization of a number of *Tr*Cel7A variants with different glycosylation patterns. Specifically, we produced both the wild-type and variants with mutated *N*-glycosylation motifs in two different expression hosts (the native *T. reesei* and the heterologous *A. oryzae*). The wide differences in the *N*-glycosylation pattern allowed us to identify important functional properties of the *Tr*Cel7A glycans.

## Results

### Thermal stability and intact protein mass spectrometry (MS) of *Tr*Cel7A and variants

**Fig. 1:**
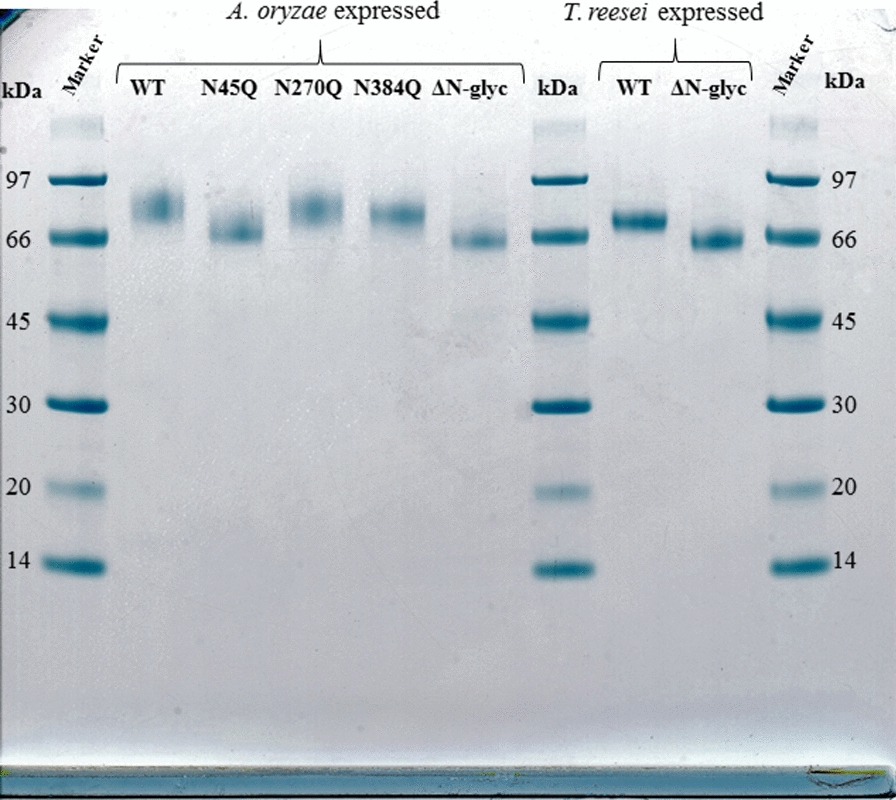
SDS-PAGE gel of the purified enzymes used in this study. Lanes 1, 7 and 10: Marker LMW (GE Healthcare) molecular weight standard; lane 2: WT_*Ao*_; lane 3: N45Q_*Ao*_; lane 4: N270Q_*Ao*_; lane 5: N384Q_*Ao*_, lane 6: ΔN-glyc_*Ao*_; lane 8: WT_*Tr*_; lane 9: ΔN-glyc_*Tr*_. The gel was stained with Coomassie Blue and 1 µg of each enzyme was loaded on the gel

In this work, we prepared and characterized a number of *Tr*Cel7A variants (Table 1). The wild-type (WT) and listed variants of *Tr*Cel7A were successfully expressed and purified as the full-length enzymes in *Aspergillus oryzae* and *Trichoderma reesei*. All the purified enzymes showed only one band in SDS-PAGE (Fig. 1). For the *Tr*Cel7A variants, the successful disruption of the *N*-glycosylation motifs through N to Q site-directed mutagenesis was verified by a decreased apparent molecular weight as shown by both SDS-PAGE (Fig. 1) and changes in the mass profiles of intact protein MS (Additional file 1: Fig. S1). At the same time, all the enzymes’ apparent MW was higher than the theoretical value of 52222 Da (see “Materials and methods” section), indicating a glycosylated protein with all functional domains. Among the *Tr*Cel7A variants expressed in *A. oryzae*, the most pronounced shift in molecular weight compared to the WT_*Ao*_ sequence was observed for the N45Q_*Ao*_, which migrated nearly as the Δ*N*-glyc_*Ao*_ (N45Q, N270Q, N384Q triple mutant).

**Table 1:** Summary of *Tr*Cel7A WT and variants with the removed *N*-glycosylation motifs by site-directed mutagenesis

*Tr*Cel7A	Mutation	Expression host	*T*_*m*_ (°C)	Residual activity (%)
WT_*Ao*_		*Aspergillus oryzae*	67.9	57 ± 1
N45Q_*Ao*_	N45Q	*Aspergillus oryzae*	67.4	38 ± 2
N270Q_*Ao*_	N270Q	*Aspergillus oryzae*	66.9	60 ± 3
N384Q_*Ao*_	N384Q	*Aspergillus oryzae*	66.1	57 ± 3
Δ*N*-glyc_*Ao*_	N45Q, N270Q, N384Q	*Aspergillus oryzae*	65.0	41 ± 2
WT_*Tr*_		*Trichoderma reesei*	67.9	45 ± 2
Δ*N*-glyc_*Tr*_	N45Q, N270Q, N384Q	*Trichoderma reesei*	65.2	44 ± 1

**Fig. 2:**
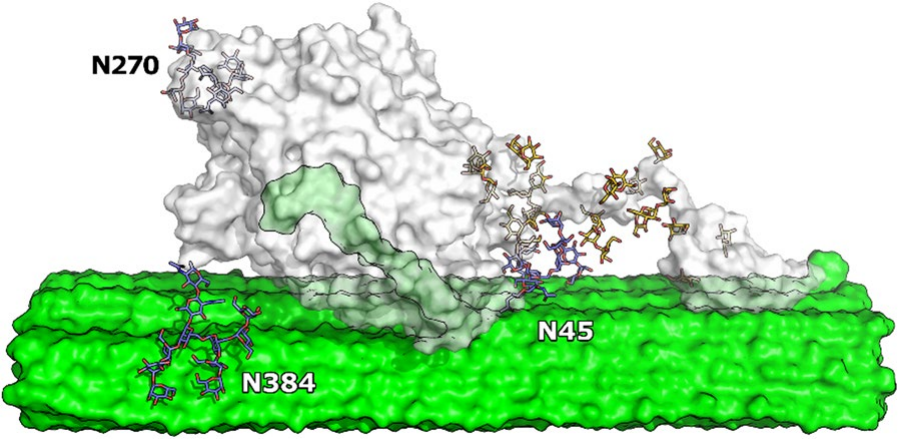
Structure of *Tr*Cel7A with labeled *N*-glycosylation sites. The structure of *Tr*Cel7A is shown as the grey surface, *N*-glycans as the blue/red sticks, *O*-glycans as the yellow/red sticks and the cellulose as the green surface. All of the experimentally determined *N*-glycosylation sites in this study are presented here: N45, N270 and N384. The cartoon representation was modeled in PyMol and it is based on the *Tr*Cel7A structure (PDB entry 4C4C) [14]. The cellulose structure was constructed using Cellulose-Builder [15]

The mutated positions of *N*-glycosylation sites are presented in Fig. 2. The thermal stability, *T*_*m*_, was determined from DSC. Residual activity is expressed as the activity after pre-incubation in the standard buffer for 1 h at *T* = *T*_*m*_− 5 °C divided by the activity of the sample incubated at 25 °C. The activity was measured on 3 mM *p*NP-Lac in 50 mM sodium acetate buffer pH 5.0 for 30 min at 25 °C

Thermal stability as measured by differential scanning calorimetry (DSC) was only moderately different among the glycovariants, and this supports the view that their overall fold was unaffected by the mutations (Additional file 1: Fig. S2). The transition temperatures of both the WTs and the ∆*N*-glyc variants were independent of the expression organism, although a small decrease in melting temperature (*T*_*m*_) was observed for the variants. The ∆*N*-glyc mutants exhibited the largest reduction of 2–3 °C in T_m_ as compared to the WT (Table 1). In case of the single mutation variants, the highest decrease in T_m_ was observed for the N384Q_*Ao*_, compared to WT_*Ao*_, which is in accordance with previous studies by Amore et al. [3] and Adney et al. [12]. Kinetic stability (residual activity at 25 °C following 1 hour exposure to a temperature equal to *T*_*m*_− 5 °C) was decreased from 57% in the wild-type to 41% in Δ*N*-glyc_*Ao*_ and N45Q_*Ao*_. For enzymes expressed in *T. reesei*, no changes were detected in the residual activity of these two forms. Finally, removal of the glycan at position N45 lowered the activity as much as the removal of all three *N*-glycans (Table 1). Moreover, neither the glycan at N270 nor N384 appeared to promote kinetic stability (unchanged residual activity for both N270Q_*Ao*_ and N384Q_*Ao*_).

Intact protein MS revealed a distinct difference in how the two expression hosts, *T. reesei* and *A. oryzae*, glycosylate the same protein (Additional file 1: Fig. S1). The WT_*Ao*_ showed a high mass heterogeneity, from which it was not possible to reconstruct the mass profile (Additional file 1: Fig. S1 D). The glycosylation pattern of WT_*Tr*_ is less heterogeneous, showing a clear mass distribution (Additional file 1: Fig. S1 A) and a lower average mass of total glycans (Table 2) as also observed by SDS-PAGE analysis (Fig. 1). Once all the *N*-glycans were removed (Δ*N*-glyc variants), it was possible to reconstruct a mass profile in all cases (Additional file 1: Fig. S1 C, E), and the remaining mass difference was mostly due to *O*-glycosylation of the linker and CBM [3]. In the case of the single *N*-glycan variants, removal of glycans at position N45 was sufficient to reconstruct the mass profile of the *Tr*Cel7A_*Ao*_ (Additional file 1: Fig. S1 F) while the other single variants retained high glycan heterogeneity (Additional file 1: Fig. S1 G, H). The estimation of mass differences for each enzyme is presented in Table 2.

**Table 2:** The extent of *N*- and *O*-glycosylation of *Tr*Cel7A WTs expressed in *T. reesei* and *A. oryzae*

*Tr*Cel7A	Molecular weight (MW) (kDa)^a^			
	Theoretical MW	MW contribution of *N*-glycosylation	MW contribution of *O*-glycosylation	Calculated MW
WT_*Tr*_	52	5–7	3–4	60–63
WT_*Ao*_	52	7–10	3–6	62–68

^a^Values were estimated based on the mass spectra presented in Additional file 1: Figure S1 A–H

### Steady-state kinetics on insoluble cellulose substrates

**Fig. 3:**
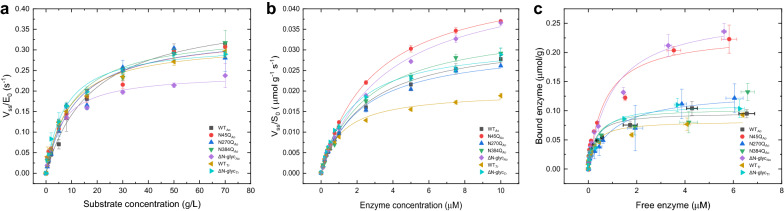
Steady-state kinetic analysis and binding isotherm for *Tr*Cel7A enzymes on Avicel at 50 °C. The reaction mixtures were incubated for 1 h in each assay. **a**^conv^MM analysis, low enzyme concentration of 0.1 μM and high Avicel load (0–70 g/L). **b**^inv^MM, low substrate concentration of 12 g/L Avicel is saturated with enzyme concentration. Solid lines are a non-linear fit from Eqs. (1,  2). **c** Binding isotherm of *Tr*Cel7A wt and variants on 12 g/L Avicel at 50 °C. Solid lines represent the fitted Langmuir equation (Eq. 4). Error bars represent standard deviations from triplicate measurements

To closer investigate the effect of glycosylation, extensive kinetic characterization of *Tr*Cel7A WTs and the variants was carried out using three insoluble cellulosic substrates, Avicel, RAC and BMCC, with widely varying physical properties (see “Materials and Methods”). To evaluate how the substrates’ properties and the changes in the *N*-glycosylation pattern influenced the kinetics, two quasi-steady-state approaches were implemented [16, 17]. The first, was the conventional Michaelis–Menten (^conv^MM) approach, using an excess of the substrate. As in usual (bulk) steady-state kinetics, this entails rate measurements with a constant and low enzyme concentration and gradually increasing substrate loads (Fig. 3a, Additional file 1: Fig. S3 A, D). Specific rates (*ν*_ss_/*E*_0_) were plotted against substrate load and non-linear regression analyses were performed accordingly to Eq. (1).

1 $${}_{{}}^{\text{conv}} v_{\text{ss}} /E_{0} = \frac{{{}_{{}}^{\text{conv}} V_{ \text{max} } S_{0} }}{{{}_{{}}^{\text{conv}} K_{M} + S_{0} }}$$

The parameters’ maximal specific rate (^conv^*V*_max_/*E*_0_) and Michaelis constant (^conv^*K*_*M*_) are found in Table 3. All the *Tr*Cel7A variants were active on the tested substrates. In contrast to previous studies [12, 18], this supports the conclusion from the DSC measurements that the investigated *N*-glycan variants retained the overall fold of Cel7A.

**Table 3:** Steady-state kinetics and binding affinity parameters (50 °C) of *Tr*Cel7A WT and the variants with modified *N*-glycosylation pattern

*Tr*Cel7A	^conv^MM			^inv^MM		Kinetic substrate accessibility	Adsorption isotherms	
	^conv^*V*_max_/E_0_	^conv^*K*_M_	*η*	^inv^*V*_max_/*S*_0_	^inv^K_M_	*Γ*_attack_	*Γ*_max_	*K*_*d*_
	(s^−1^)	(g/ L)	(L/g/s)	(μmol/g/s)	(μM)	(μmol/g)	(μmol/g)	(μM)
Avicel								
WT_*Ao*_	0.40 ± 0.01^a^	18.1 ± 1.8^a^	0.022	0.034 ± 0.001	2.45 ± 0.18	0.08 ± 0.00	0.1 ± 0.01	0.26 ± 0.06
N45Q_*Ao*_	0.34 ± 0.02	11.6 ± 2.5	0.030	0.048 ± 0^a^	2.92 ± 0.04^a^	0.14 ± 0.01	0.23 ± 0.02^a^	0.69 ± 0.21
N270Q_*Ao*_	0.35 ± 0.02	11.6 ± 2.4	0.030	0.031 ± 0.001	2.22 ± 0.2	0.09 ± 0.01	0.13 ± 0.01	1 ± 0.28
N384Q_*Ao*_	0.35 ± 0.02	10.6 ± 1.5	0.033	0.037 ± 0.001^a^	2.54 ± 0.25^a^	0.11 ± 0.01	0.10 ± 0.01	0.3 ± 0.18
Δ*N*-glyc_*Ao*_	0.24 ± 0.01^a^	6.2 ± 0.8	0.040	0.048 ± 0.002^a^	3.54 ± 0.3^a^	0.20 ± 0.01	0.27 ± 0.02^a^	1.01 ± 0.22^a^
WT_*Tr*_	0.32 ± 0.01	9 ± 1.2	0.035	0.02 ± 0.001^a^	1.16 ± 0.09^a^	0.06 ± 0.00	0.08 ± 0.01^a^	0.27 ± 0.08
Δ*N*-glyc_*Tr*_	0.32 ± 0.01	7.4 ± 0.9	0.043	0.032 ± 0.001	1.71 ± 0.12	0.10 ± 0.00	0.11 ± 0.01	0.4 ± 0.11
RAC								
WT_*Ao*_	0.48 ± 0.02^a^	1.3 ± 0.2^a^	0.37	0.65 ± 0.01	0.95 ± 0.06	1.4 ± 0.1	0.92 ± 0.04^a^	0.03 ± 0.01
N45Q_*Ao*_	0.37 ± 0.02	0.9 ± 0.1	0.41	0.74 ± 0.02^a^	1.23 ± 0.1^a^	2.0 ± 0.1	1.79 ± 0.0 ^a^	0.05 ± 0^a^
N270Q_*Ao*_	0.38 ± 0.02	1.1 ± 0.2	0.33	0.61 ± 0.01	1.15 ± 0.08	1.6 ± 0.1	1.14 ± 0.05^a^	0.03 ± 0.01
N384Q_*Ao*_	0.34 ± 0.01^a^	0.8 ± 0.1	0.43	0.62 ± 0.02	1.03 ± 0.08	1.8 ± 0.1	1.2 ± 0.08^a^	0.02 ± 0.01
Δ*N*-glyc_*Ao*_	0.30 ± 0.01^a^	0.5 ± 0.0^a^	0.40	0.68 ± 0.01^a^	0.82 ± 0.05	2.9 ± 0.1	1.58 ± 0.04	0.11 ± 0.01^a^
WT_*Tr*_	0.47 ± 0.01^a^	1 ± 0.1	0.45	0.46 ± 0.01^a^	0.94 ± 0.09	1.0 ± 0.0	1.83 ± 0.18^a^	0.01 ± 0.01^a^
Δ*N*-glyc_*Tr*_	0.47 ± 0.01^a^	0.8 ± 0.1	0.58	0.59 ± 0.02	0.93 ± 0.1	1.3 ± 0.0	1.59 ± 0.16	0.03 ± 0.01
BMCC								
WT_*Ao*_	1.46 ± 0.14^a^	2.1 ± 0.4^a^	0.71	0.45 ± 0.02^a^	1.59 ± 0.15^a^	0.31 ± 0.03	1.17 ± 0.12	1.28 ± 0.29
N45Q_*Ao*_	1.33 ± 0.09^a^	1.7 ± 0.2^a^	0.77	0.52 ± 0.02^a^	1.23 ± 0.11	0.39 ± 0.03	1.96 ± 0.35^a^	1.2 ± 0.44
N270Q_*Ao*_	1.13 ± 0.06^a^	1.1 ± 0.1	1.01	0.44 ± 0.02	1.49 ± 0.14^a^	0.39 ± 0.03	0.93 ± 0.15^a^	0.99 ± 0.38
N384Q_*Ao*_	1.01 ± 0.03	1 ± 0.1	1.02	0.38 ± 0.02	1.29 ± 0.15	0.38 ± 0.02	1.06 ± 0.04	0.85 ± 0.08
Δ*N*-glyc_*Ao*_	0.96 ± 0.09	1.6 ± 0.3^a^	0.59	0.48 ± 0.01^a^	1.38 ± 0.1^a^	0.50 ± 0.05	1.2 ± 0.06	0.62 ± 0.09
WT_*Tr*_	0.63 ± 0.03^a^	0.7 ± 0.1	0.89	0.29 ± 0.01^a^	0.66 ± 0.06^a^	0.46 ± 0.03	1.34 ± 0.11	0.48 ± 0.12
Δ*N*-glyc_*Tr*_	0.76 ± 0.03	0.6 ± 0.1^a^	1.33	0.4 ± 0.01	0.82 ± 0.06^a^	0.53 ± 0.03	1.71 ± 0.04^a^	0.56 ± 0.04

The parameters were derived from ^conv^MM, ^inv^MM and binding isotherm using Avicel, RAC and BMCC. The ± values correspond to the error of non-linear fit of Michaelis–Menten curves and binding isotherm curves. The parameters statistically different from the others at the 0.05 level of significance are indicated with letter ‘a’ (Additional file 1: Table S2)

The ^conv^MM analysis (Table 3) demonstrated a correlation between *K*_*M*_ and the degree of glycosylation. Thus, a reduction in the degree of *N*-glycosylation was consistently associated with lowered *K*_*M*_. This was particularly evident on Avicel and BMCC, and it appeared both when comparing WTs and Δ*N*-glyc variants and with the WT expressed in different hosts (i.e. WT_*Tr*_ exhibited a lower *K*_*M*_ as compared to the more extensively glycosylated WT_*Ao*_, c.f. Table 2).

In the well-known ^conv^MM approach, saturation represents the situation where all enzymes are complexed with the substrate, and the saturation rate hence reflects the maximal turnover (*k*_cat_ = ^conv^*V*_max_/*E*_0_). In the current work, steady-state kinetics in the opposite limit was also investigated, where the enzyme is in excess (inverse Michaelis–Menten, ^inv^MM). In this approach, measurements were made with 1 h reaction rates at a constant, low substrate load and gradually increasing enzyme concentration. This method has been reported [19] to be specifically useful for heterogeneous systems because a steady-state condition may occur also when the enzyme is in excess. Derivation of the associated rate equation (Eq. 2) [20, 21] as well as assumptions and limitations of this approach have been discussed elsewhere [17, 19].

2 $${}_{{}}^{\text{inv}} v_{\text{ss }} / S_{0} = \frac{{{}_{{}}^{\text{inv}} V_{ \text{max} } E_{0} }}{{{}_{{}}^{\text{inv}} K_{M} + E_{0} }}$$

In the ^inv^MM approach, saturation represents the situation where all attack sites on the surface of the insoluble substrate are complexed with an enzyme, and the maximal rate hence represents the product of the maximal turnover and the density of attack sites, ^inv^*V*_max_/*S*_0_ = *k*_cat_*Γ*_attack_, where *S*_0_ is the mass load of a substrate and *Γ*_attack_ the number of sites per mass unit of substrate [17].

It follows that one may calculate the density of attack sites *Γ*_attack_ (also named productive binding capacity [22]) as the ratio of two specific maximal rates as shown in Eq. (3).

3 $$\varGamma_{\text{attack}} = \frac{{{}_{{}}^{\text{inv}} V_{ \text{max} } /S_{0} }}{{{}_{{}}^{\text{conv}} V_{ \text{max} } /E_{0} }}$$

The data points obtained from the ^inv^MM measurements (Fig. 3b and Additional file 1: Fig. S3 B, E) were subjected to the best fit from non-linear regression (Eq. 2). Based on this analysis, the parameters, as well as *Γ* attack values (Eq. 3), were calculated (Table 3). These results also show some interesting systematic trends. In particular, *Γ*_attack_ is increased in variants that do not have the glycan in position N45. This is true for both the N45Q_*Ao*_ and the ∆*N*-glyc_*Ao*_ and ∆*N*-glyc_*Tr*_. Removal of the other two *N*-glycans (N270Q_*Ao*_ and N384Q_*Ao*_) also improved the enzyme’s ability to “find” attack sites on the cellulose surface, but the effect was less pronounced.

### Binding isotherms

Surface coverage (*Γ*, in µmol/g) was derived from measurements of free enzyme concentration in 1-h equilibrated enzyme–substrate suspensions. Cruys-Bagger et al. [23] presented that equilibrium state on bacterial cellulose was achieved after 10 min and it is assumed the same for other cellulosic substrates used here. The experimental data were fitted to a simple Langmuir isotherm (Eq. 4) (Fig. 3c and Additional file 1: Fig. S3 C, F). While the adsorption mechanism of Cel7A is complex [24, 25], simple Langmuir parameters have often been used for comparative purposes.

4 $$\varGamma \; = \;\frac{{\varGamma_{ \text{max} } E_{\text{free}} }}{{K_{d} E_{\text{free}} }}$$

The maximal adsorption capacities (*Γ*_max_) on Avicel, RAC and BMCC were higher for the N45Q_*Ao*_ and Δ*N*-glyc_*Ao*_ relative to WT_*Ao*_ (Table 3). As the *Γ*_max_ varied only moderately for the other two variants (N270Q_*Ao*_ and N384Q_*Ao*_), elimination of the *N*-glycan at position N45 seems to promote surface coverage on all cellulosic substrates. For the *Tr*Cel7A expressed in *T. reesei*, the difference in *Γ*_max_ between WT_*Tr*_ and Δ*N*-glyc_*Tr*_ was much lower than for the same enzyme pair expressed in *A. oryzae*.

*Γ*_max_ and *Γ*_attack_ are related parameters in as much as they both quantify a site density on the cellulose surface. However, they differ as *Γ*_max_ enumerates all sites (productive and non-productive) whereas *Γ*_attack_ specifically describes productive sites. Despite this difference, the two parameters are quite similar and show the same tendencies with respect to glycan dependence (Table 3).

### Molecular dynamics (MD) simulations of *Tr*Cel7A *N*-glycan variants on cellulose crystals

**Fig. 4:**
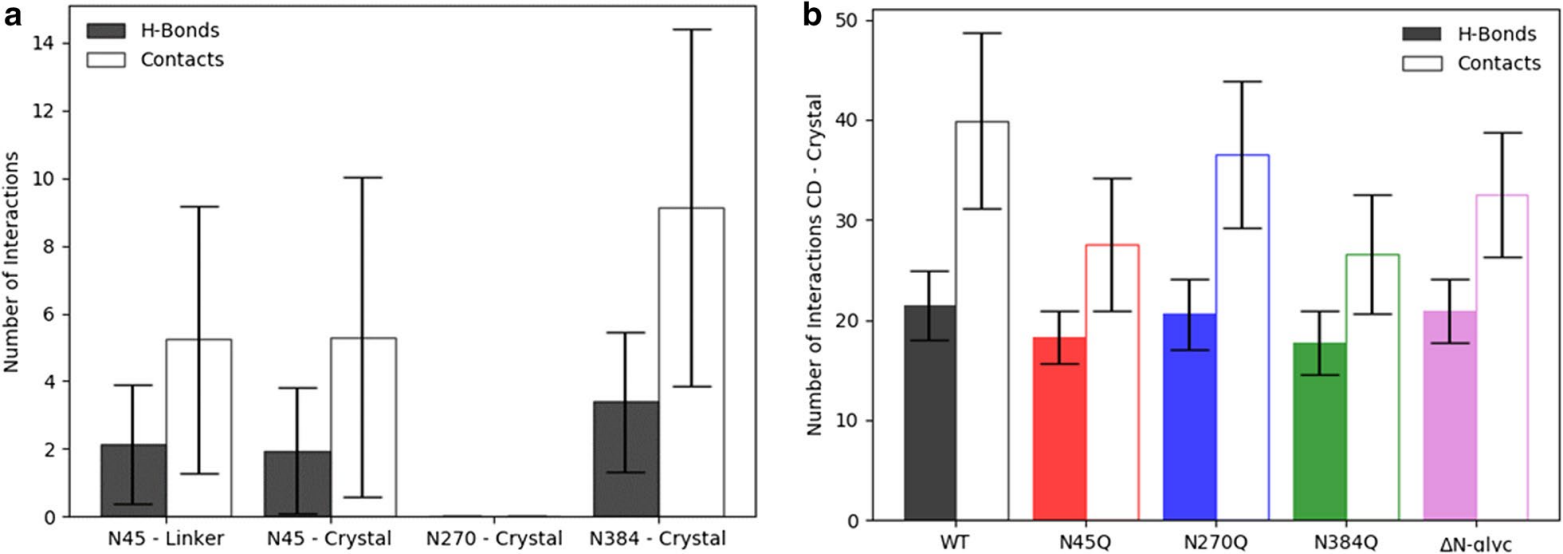
Quantification of *Tr*Cel7A structure binding to cellulose crystal surface by 0.1-µs molecular simulations. The filled and unfilled bars show the average number of contact and hydrogen bond pairs, respectively, and the bars the corresponding standard deviation over the course of the simulation. **a** Interaction pairs formed between *N*-glycans of CD WT and cellulose crystal surface as well as between N45 glycan and linker *O*-glycans. **b** Interaction pairs formed between CD of the *N*-glycan knockouts and the cellulose crystal

To further elucidate the role of *N*-glycosylation in *Tr*Cel7A, 0.1-µs MD simulations of the WT *Tr*Cel7A and the corresponding *N*-glycan enzyme variants productively bound to cellulose crystal were initiated. The *N*- and *O*- glycosylation pattern was built as described in “Materials and methods” section. From the MD data, the number of contacts and hydrogen bonds between *N*-glycans and cellulose crystal surface was calculated. The glycans at position N45 and N384, but not N270, interacted with the cellulose surface (Fig. 4a). The glycans at N384 exhibited a higher number of contacts and hydrogen bonding compared to N45. Interestingly, the glycans at N45 engaged in a similar number of contacts and hydrogen bonds with linker *O*-glycans. The numbers of hydrogen bonding and contact pairs between the series of *N*-glycan variants and the cellulose crystals were calculated (Fig. 4b). The removal of glycans at either N45 or N384 decreased the number of contacts between the CD and cellulose crystal. The minor increase in the number of contacts for the Δ*N*-glyc variant corresponded to the improved peptide–crystal interaction. Therefore, the molecular simulations indicated that *N*-glycans can be involved in the modulation of *Tr*Cel7A kinetics due to the possible interactions with cellulose surface.

## Discussion

Cellobiohydrolases from Glycoside Hydrolase family 7 make up a major part of enzyme cocktails for the industrial deconstruction of lignocellulosic biomass. The enzyme from *T. reesei*, *Tr*Cel7A, is the most studied GH7, and like other fungal GH7 cellobiohydrolases, it carries several *N*-glycans on its CD. The functional roles of these *N*-glycans have been widely discussed and they have been linked to both thermal stability and protease resistance [3]. They have also been associated with catalytic properties including lower cellulose conversion of hyperglycosylated enzymes and higher activity of enzymes with single deletions of *N*-glycosylation sites [12, 18]. As the position, pattern and heterogeneity of fungal enzyme glycosylation depend on numerous factors [6, 11], it is not straightforward to assess the structure–function relationships based on wild-type enzymes. Therefore, comparative biochemistry of glycovariants appears necessary to elucidate interrelationships of *N*-glycosylation and catalytic performance. To implement this strategy, a comparative biochemical study of *Tr*Cel7A variants with different *N*-glycosylation patterns was conducted. Specifically, two industrially relevant different expression hosts were evaluated and enzymes with different *N*-glycan knockouts and substrates with widely differing physical properties were tested. The results provided a wide range of kinetic and binding data and some general trends on the functional roles of *N*-glycans emerged.

A consistent tendency for the Michaelis constant, ^conv^*K*_*M*_, to increase with the degree of glycosylation was observed, as illustrated by the kinetics on Avicel of the *N*-glycan variants expressed in *A. oryzae* (Table 3). In this series, WT_*Ao*_ had a ^conv^*K*_*M*_ of 18 g/L. Disruption of one of the glycosylation motifs in either of the variants N45Q_*Ao*_, N270Q_*Ao*_ or N384Q_*Ao*_ lowered this value significantly, and for the Δ*N*-glyc_*Ao*_, it was reduced even further to 6 g/L. A similar trend was observed for RAC and BMCC (Table 3). Moreover, the propensity of ^conv^*K*_*M*_ to scale with the degree of glycosylation also emerged when comparing the WT sequence expressed in different hosts. A lower ^conv^*K*_*M*_ was observed for the enzymes from *T. reesei* which were less glycosylated than the enzymes from *A. oryzae* [5, 12] (Fig. 1, Additional file 1: Fig. S1). In the same ^conv^MM approach, the ∆*N*-glyc_*Ao*_ variant exhibited consistently lower maximal specific rate, ^conv^V_max_/E_0_ (*k*_cat_), in respect to the WT_*Ao*_. This was observed with all three cellulosic substrates: 0.4 vs. 0.24 s^−1^ on Avicel; 0.48 vs. 0.30 s^−1^ on RAC and 1.46 vs. 0.96 s^−1^. Inverse Michaelis–Menten showed systematic effects linked to the degree of glycosylation. Attack site density (*Γ*_attack_) was found to vary distinctively between the substrates as demonstrated earlier [26] with RAC and BMCC being more accessible with more attack sites compared to Avicel. Moreover, *Γ*_attack_ elucidates the enzyme’s ability to engage in productive complexes on the cellulose surface and it was found to be systematically related with the extent of glycosylation (Table 3). With Avicel as an example, the WT_*Ao*_ had *Γ*_attack_ = 80 nmol/g cellulose. In variants with disruption of one glycosylation motif, the value ranged from 90 to 140 nmol/g cellulose, and for the ∆*N*-glyc_*Ao*_ it was 200 nmol/g cellulose. In other words, ∆*N*-glyc_*Ao*_ recognized more than twice as many productive binding sites on Avicel as compared to the WT. The inverse relationship of glycosylation and *Γ*_attack_ was also evident for the two other substrates (RAC and BMCC), irrespective of the production host (Table 3). The same trend was observed in the independently measured adsorption data in Table 3. Thus, the saturation coverage, *Γ*_max_, was generally increased by disruption of *N*-glycosylation motifs. Interestingly, WT_*Ao*_ and WT_*Tr*_, did not show identical *Γ*_attack_ on each tested substrate, and this might be attributed to the overall glycosylation differences between these two enzymes (Table 2). In particular, this could be linked to changes in *O*-glycosylation of *Tr*Cel7A, which has been shown to be involved in cellulose binding [27, 28].

The systematic changes in both ^conv^*K*_*M*_ and *Γ*_attack_ are most likely interlinked. Thus, an enzyme that recognizes many attack sites (high *Γ*_attack_) requires less substrate (in g/L) to reach a reaction rate that is half of ^conv^*V*_max_. In other words, the apparent molar concentration of attack sites of substrates increases with *Γ*_attack_ and it follows that enzyme–substrate systems with the high *Γ*_attack_ will tend to have low ^conv^*K*_*M*_. One possible explanation for the improved binding affinity and heightened ability to find more attack sites on the cellulose surface is that glycan structures impose a steric hindrance, which hampers the enzyme from achieving an optimal conformation on the cellulose surface. In other words, the *N*-glycans may interfere with the enzyme’s association to the cellulosic substrates. The glycans at N45 and N384 are located in close distance to the entrance and exit of the catalytic tunnel, respectively (Fig. 2). Therefore, glycans in both positions could be involved in modulating enzyme–substrate association. It has been suggested by Adney et al. [12] that the glycans at N384 may act as a “spacer”, limiting access to the substrate by increasing the distance between the catalytic domain and cellulose surface. A similar observation of the negative impact of N45 glycan on Cel7A from *Penicillium verruculosum* was reported by Dotsenko et al. [18]. This interpretation can be further linked to the results of the MD simulation of *Tr*Cel7A WT and the corresponding *N*-glycan variants. MD simulations demonstrated that both glycans at N45 and N384 interacted with the cellulose crystal providing multiple contact pairs and some hydrogen bond pairs. No interactions were found for the glycans at N270, most likely due to the distal position to the cellulose crystal (Fig. 4a). Simulations including the mutations N45Q and N384Q showed a lower number of contacts between *Tr*Cel7A CD and cellulose surface compared to *Tr*Cel7A CD with intact glycans (Fig. 4b). Therefore, the higher number of contacts enabled by the presence of *N*-glycans may prevent catalytic peptide–cellulose interactions. This would result in an apparent lowered cellulose affinity of *Tr*Cel7A and the frequency of finding productive binding sites. Moreover, a number of contacts were found between the glycans at N45 and linker *O*-glycans (Fig. 4a), which supported the view of Jeoh et al. [5] and Adney et al. [12] that *N*-glycans could potentially interact with linker and CBM domains of *Tr*Cel7A. However, we found that removal of *N*-glycans from the catalytic domain of *Tr*Cel7A (without linker and CBM) by endoglycosidase H treatment, still resulted in improved binding capacity and activity within ^inv^MM conditions on Avicel (Additional file 1: Fig. S4). These results are consistent with the trend observed for Δ*N*-glyc *Tr*Cel7A that *N*-glycosylation is more important for substrate interactions compared to interactions between the enzyme’s domains.

More detailed inspection of the kinetic data in Table 3 suggested that the three *N*-glycan variants varied with respect to their influence on enzyme function. In particular, it appeared that the glycans at N45 exerted the most pronounced effect on the kinetic- and binding parameters. N45 is located on a loop region preceding the B1 loop (Additional file 1: Fig. S5) [29], in proximity to the entrance of the catalytic tunnel (Fig. 2). The *N*-glycans at this position can have various degree of polymerization, varying from a single GlcNAc to high mannose type structures [3, 11]. However, in this study, WT_*Ao*_ seems to only have glycans with high molecular weight and heterogeneity (Fig. 1, Additional file 1: Fig. S1). The strong correlation between the attack site density (*Γ*_attack_) and maximal adsorption capacities (*Γ*_max_) (Fig. 3c, Table 3) shows that the N45 *N*-glycans not only hinder the ability to form productive complexes on the cellulose surface, but it also decreases the overall adsorption. The negative impact of the N45 *N*-glycans on *Γ*_max_ and *Γ*_attack_ could be further explained by the fact that *Tr*Cel7A loops, shaping the catalytic tunnel, play a key role in modulating the enzyme’s ability to attack cellulose surface [30] and kinetics [31]. Thus, a bulky, heterogeneous glycan structure on the entrance loop might lead to reduced productive binding on the cellulose surface.

Moreover, the decrease in *V*_max_/E_0_ observed for the ∆*N*-glyc_*Ao*_ is well correlated with the higher substrate affinity (lower *K*_*M*_) on all of the three cellulosic substrates. This could be translated to the fact that the enzyme has a higher capability to achieve a tighter binding to the cellulose surface which might lead to a slower dissociation from the substrate. Since the rate-limiting step for TrCel7A is governed by dissociation constant (*k*_off_) [31], the removal of *N*-glycans might further decrease it.

The described kinetic trends for *Tr*Cel7A upon removal of *N*-glycans were also observed when tested on two different cellulosic substrates, RAC and BMCC which represent model substrates with low (CrI < 0.05 [33, 34]) and high (CrI ~ 0.92 [34]) crystallinity index, respectively. Avicel has a moderate crystallinity index (CrI ~ 0.6 [2, 33]). As the general trend of lower K_M_ and higher *Γ*_attack_ was maintained for all three substrates (Table 3), this suggests that the overall effect of *N*-glycans on the substrate interactions was independent of crystallinity. In accordance with RAC being an easily accessible cellulosic substrate with a high number of potential attack sites, *Γ*_attack_ was found to be the highest on RAC. This result is accordance with previous work by Nill and Jeoh [26] in which showed that swollen cellulose, prepared similarly to RAC, had the highest productive binding capacity among the tested model cellulosic substrates.

It is important to emphasize that *N*-glycosylation motifs are not well conserved among the Cel7A catalytic domains in family GH7 (Additional file 1: Fig. S6). This might be correlated with the habitat of the microorganisms secreting these enzymes, the likelihood of horizontal gene transfer, and environmental pressure. Depending on the benefits conferred by the glycosylation, variability in *N*-glycan positions could result in higher stability or lower substrate affinity in Nature but they might not be desirable for the in vitro conditions. We propose that functional assessments of glycosylation require a broad characterization based on comparative biochemistry and the current work suggests one approach to this. We found that *Tr*Cel7A kinetics and binding affinity can be modulated by changing *N*-glycosylation composition achieved by site-directed mutagenesis, selection of expression hosts and *N*-glycan specific glycosidases. Recently, Rubio et al. [35] presented a study in which GH3 β-xylosidase activity was enhanced by a new *N*-glycosylation design. We believe that our kinetic toolbox can be applied to other biomass-degrading enzymes and to our knowledge, this is the first study showing an in-depth kinetic characterization *Tr*Cel7A with modified *N*-glycosylation pattern, tested on different insoluble cellulosic substrates.

## Conclusions

Detailed kinetic analysis of a high number of enzymes with modified *N*-glycosylation pattern revealed additional roles in modulation of activity and binding properties of *Tr*Cel7A on insoluble cellulosic substrates. The removal of *N*-glycans from *Tr*Cel7A lowered the conventional Michaelis constant (*K*_*M*_), increased the kinetic substrate accessibility (*Γ*_attack_) and saturation coverage (*Γ*_max_). In particular, the absence of glycans at N45 modulated ability of the Cel7A to find a higher amount of productive attack sites. Both *Tr*Cel7A WT and the variant Δ*N*-glyc exhibited the same kinetic trends regardless of the glycosylation patterns conferred by the used expression host, *A. oryzae* or *T. reesei*. The interrelationships between *N*-glycosylation and substrate binding may reflect steric hindrance which encumbers formation of the optimal conformation for substrate attack. This was further emphasized by the molecular simulations, which illustrated the capability of the *N*-glycosylation to interact with cellulose surface. Our results provide functional and structural insights into *N*-glycosylation of GH7 *Tr*Cel7A that can be further utilized for better understanding of this industrially relevant cellulose-degrading type of enzyme. Potentially, the findings could form a basis for predicting the effect of glycosylation for other interfacial-active enzymes.

## Materials and methods

### Enzymes

*Trichoderma reesei* Cel7A (*Tr*Cel7A) enzymes were cloned and expressed in *Aspergillus oryzae* as described earlier [36]. The changes in *N*-glycosylation were introduced by point mutations of asparagine to glutamine in the *N*-glycosylation motifs. Four *Tr*Cel7A mutants were generated: (1) N45Q, (2) N270Q, (3) N384Q and combination of all three mutations (4) (N45Q, N270Q, N384Q). Enzyme purification was performed according to the protocol also described previously [37]. *T. reesei* expressed *Tr*Cel7A WT and Δ*N*-glyc were supplied as purified enzymes from Novozymes A/S. Protein concentration was determined by absorbance measurement at 280 nm using a theoretical molar extinction coefficient 81930 M/cm derived from the amino acid sequence without signal peptide. Theoretical molecular weight and extinction coefficient were calculated based on the amino acid sequence without signal peptide from P62694 (UniProt entry). The enzyme purity was verified by SDS-PAGE using NuPAGE 4–12% Bis–Tris gels (GE Healthcare, Fig. 1) and molecular weight was estimated by a reference to the commercial marker kit (LMW, GE Healthcare).

### Intact protein mass spectrometry

All the studied enzymes were analyzed for their intact molecular weight using a MAXIS II electrospray mass spectrometer (Bruker Daltonik GmbH, Bremen, Germany). The samples were diluted to 0.1 mg/mL and applied to an AdvanceBio Desalting-RP column (Agilent Technologies). Samples were eluted from the column with an acetonitrile linear gradient from 5 to 95% (v/v) and introduced to the electrospray source with a flow of 400 mL/min by an Ultimate 3000 LC system (Thermo Fisher Scientific). Data analysis was performed with DataAnalysis version 4.3 (Bruker Daltonik GmbH, Bremen, Germany).

### Substrates

Enzyme activity and binding affinity were measured on three different cellulosic substrates: regenerated amorphous cellulose (RAC), Avicel PH101 (Sigma-Aldrich, St. Louis, MO) and bacterial microcrystalline cellulose (BMCC). RAC was prepared from Avicel as described earlier [39, 40]. BMCC was prepared from bacterial cellulose (BC) as described in [34, 35]. All substrates were washed in MiliQ water and then washed and stored in 50 mM sodium acetate buffer pH 5.0 (referred to as a standard buffer) in the presence of 5 mM sodium azide.

### Steady-state kinetics

Enzymes’ kinetics were measured at the two different experimental settings: enzyme saturation (conventional Michaelis–Menten; ^conv^MM) and substrate saturation (inverse Michaelis–Menten; ^inv^MM). In ^conv^MM conditions, 230-μL substrate aliquots, from a homogenous vigorously stirred cellulosic substrate stock, with various concentration were pipetted into 96-well microtiter plate (96F 26960 Thermo Scientific, Waltham, MA). The hydrolysis reaction was initiated by adding 20 μL enzyme with to a final concentration of 100 nM when tested with Avicel and 50 nM with RAC and BMCC. In the ^inv^MM conditions, 190 μL substrate with fixed concentration (12 g/L Avicel, 0.4 g/L RAC, 0.75 g/L BMCC) was pipetted into 96-well microtiter plate. The reactions were initiated by adding 60-μL enzyme aliquots of different concentration. The enzymatic reactions were performed for 1 h at either 25 or 50 °C, mixed at 1100 rpm and then quenched by centrifugation for 3 min at 2500*g.* From each reaction, 60 μL of supernatant was collected and mixed with 90 μL of *p*-hydroxybenzoic acid (PAHBAH) to quantify released reducing sugar ends [40]. The detailed experimental procedure is described elsewhere [37]. The absorption of the colored products was measured at 405 nm using a plate reader (Spectra Max 3; Molecular Devices, Sunnyvale, CA, USA). The absorbance readouts were recalculated to reducing sugar ends’ concentration using a cellobiose standard curve (0–1 mM). The obtained reaction rate curves were fitted with Eqs. (1, 2) for ^conv^MM and ^inv^MM conditions, respectively. The fitting was done in Origin Pro (version 2019, OriginLab Corporation, Northampton, MA, USA). Each reaction was performed in triplicate.

### Binding isotherms

Enzyme adsorption on cellulose substrates was measured using the same experimental setup as for the ^inv^MM conditions. 60 μL of supernatant was transferred to 96-well microtiter plate (655079, Greiner Bio One) and mixed with 90 μL standard buffer. The enzyme concentration was measured by intrinsic protein fluorescence at 280 nm excitation wavelength and 345 nm emission wavelength. The free enzyme concentration was quantified using the calibration curve composed of known enzyme concentrations diluted in the standard buffer. Reactions were performed in triplicate measurements. The results were fitted to the Langmuir isotherm shown in Eq. (3). Maximal adsorption capacity (*Γ*_max_) and dissociation constant (*K*_*d*_) were estimated and the binding affinity for each studied enzyme was analyzed.

### Analysis of means

To compare the parameters derived from steady-state kinetic and binding isotherm experiments, the datasets were modeled in JMP Pro 15 (version 2019, SAS Institute Inc., Cary, NC, 1989–2019) according to the corresponding equations. The obtained parameters within one dataset were compared using analysis of means. Based on the analysis of means plots (Additional file 1: Figure), the parameters statistically different from the others at the 0.05 level of significance are indicated with a letter ‘a’.

### Thermal stability and residual activity

The studied enzymes were analyzed by differential scanning calorimetry (DSC, MicroCal VP-Capillary DSC from Malvern Panalytical). The enzymes were buffer exchanged using desalting columns (PD MidiTrap G-25, GE Healthcare Life Sciences) to 50 mM sodium acetate buffer pH 5.0 and further diluted to a concentration of 0.5 mg/mL. Thermal stability was tested with a heating scan range from 20 to 100 °C and a scan rate of 3.3 °C/min. Buffer scans were subtracted from enzyme scans. Thermal transitions were observed only for the enzyme samples. Data were collected and analyzed with Origin 7 software (OriginLab, Northampton, MA, USA). As a result, the transition midpoints (*T*_*m*_) were obtained for each of the enzymes.

Residual activity of *Tr*Cel7A enzymes was measured on *para*-nitrophenyl β-d-lactopyranoside (*p*NPL) in 50 mM sodium acetate pH 5.0. Prior to the activity assay, the enzymes at the concentration of 0.5 μM were incubated at the temperature *T = T*_*m*_− 5 °C for 1 h. Then, the activity assay was initiated by adding the enzyme to *p*NPL, with the assay concentration of 0.2 μM and 0.5 mM, respectively. After 30 min, the reaction was quenched with 150 μL of 1 M Na_2_CO_3_. 150 μL of the quenched reaction mixture was transferred to a new microtiter plate and the absorbance at 405 nm was measured. The concentration of released *para*-nitrophenol (*p*NP) was quantified against the standard curve made with known concentrations of *p*NP (15–1000 μM). Each reaction was performed in triplicate measurements. The residual activity was calculated by comparing the activities of the enzyme with and without thermal pre-incubation.

### Molecular simulations

The structure of the CD was taken from PDB [41] entry 4C4C [14]. In the WT structure, all three *N*-glycosylation sites (N45, N270, and N384) were decorated with (Man)_9_(GlcNAc)_2_ using the doGlycans program [42]. The structure of the CBM was taken from PDB entry 2CBH [43]. The linker and the CBM were decorated as discussed by Harrison et al. [10]. A cellulose fibril of 15 cellobiose units in length was constructed using the Cellulose-Builder [15]. The general placement of the enzyme was done similar to work by Payne et al. [44]. The lower half of the fibril was removed to limit the simulation box size. A cellulose chain on the edge of the fibril was partly removed and the CD was placed in a way that the bound cellononaose could be linked to the remaining part of that cellulose chain. Besides the wild-type structure, *Tr*Cel7A enzyme variants were prepared (N45Q, N270Q, N384Q, and a Δ*N*-glyc variants).

The structure was taken and run through the CHARMM-GUI [45] to obtain a GROMACS topology. The CHARMM36 force field was used to describe the system [48, 49]. All simulations were run in GROMACS 2018.6 [48–55]. GROMACS was used to construct a triclinic box with a minimal distance of 10 Å and solvate the system with TIP3P water [58, 59]. To neutralize the net charge of the system, random water molecules were exchanged with sodium ions. Minimization was done in a steepest-descent over 10,000 iterations. Afterward, NVT simulations with incremental temperature (100 K to 300 K in 50 K steps) were performed in succession for 20 ps each. Thereafter, NPT simulations with restraints on all solutes, with restraints on the protein backbone and the crystal, and with restraints only on the crystal were performed for 100 ps in series. The production was run in the NPT ensemble at 300 K with a time-step of 2 fs for 100 ns, while only keeping the lowest layer of cellulose chains in the crystal restraint. The long-range electrostatic treated with the Particle-mesh Ewald method with cubic interpolation and a cut-off of 12 Å [58]. Van der Waals interactions were treated in a Verlet scheme with a cut-off distance of 12 Å and a switching function for the forces starting at 10 Å [59]. Bonds were restrained using the LINCS algorithm [60]. The solutes and the solvent were coupled to heat baths at 300 K with a Berendsen thermostat [61]. Pressure coupling was done with a Parrinello–Rahman barostat [62]. Analysis of the trajectories was performed with GROMACS. The trajectories were visualized in PyMOL.

### Multiple sequence alignment and phylogenetic tree

To construct the phylogenetic tree, 29 characterized GH7 Cel7A amino acid sequences were retrieved from Carbohydrate Active Enzymes (CAZy) database (http://www.cazy.org/) [8] and the multiple sequence alignment was performed using ClustalW [63]. As the reference sequence, *Trichoderma reesei* Cel7A was selected. The gap-opening values of the pairwise and multiple alignments were set to 3 and 30, respectively, while the other parameters were kept default. The amino acid residues corresponding to signal peptide, linker and CBM were removed from the aligned sequences and subsequently realigned. The phylogenetic analysis was done using MEGA X [64]. The phylogenetic tree was built using the Maximum Likelihood method and Whelan and Goldman model [65]. The tree with the highest log likelihood (− 12018.02) is shown. Initial tree(s) for the heuristic search was obtained automatically by applying Neighbor-Join and BioNJ algorithms to a matrix of pairwise distances estimated using a JTT model, and then selecting the topology with superior log likelihood value. All positions with less than 90% site coverage were eliminated, i.e., fewer than 10% alignment gaps, missing data, and ambiguous bases were allowed at any position.

## Supplementary information

**Additional file 1: Fig. S1.** Mass distribution profile of full-length TrCel7A from intact protein MS. **Fig. S2.** DSC scans of all TrCel7A enzymes used in this study. **Table S1**. Steady-state kinetics and binding affinity parameters (25 °C) of TrCel7A WT and the variants with modified *N*-glycosylation pattern. **Fig. S3.** Steady-state kinetic analysis and binding isotherm for TrCel7A enzymes on RAC and BMCC at 50 °C. **Fig. S4.**^Inv^MM analysis and binding isotherm of TrCel7A CD WT and endoH treated TrCel7A CD on 12 g/L Avicel. **Fig. S5**. An architecture-based amino acid sequence of the Trichoderma reesei Cel7A. **Fig. S6.** Phylogenetic tree of the characterized Cel7A sequences from GH7 (CAZy database).** Table S2**. Statistical comparison by analysis of means of the parameters derived from non-linear fit of Michaelis-Menten and binding isotherms curves (50 °C). **Table S3**. Statistical comparison by analysis of means of the parameters derived from non-linear fit of Michaelis-Menten and binding isotherms curves (25 °C).

## Data Availability

The datasets supporting the conclusions of this article are included within the article and in Additional file 1.

## Abbreviations

*Tr*Cel7A: *Trichoderma reesei* Cel7A
CAZy database: Carbohydrate-active enzyme database
CD: Catalytic domain
CBM: Carbohydrate-binding module
Man: Mannose
GlcNAc: *N*-acetylglucosamine
MS: Mass spectrometry
SDS-PAGE: Sodium dodecyl sulfate–polyacrylamide gel electrophoresis
MW: Molecular weight
DSC: Differential scanning calorimetry
Tm: Melting temperature
*p*NP-Lac: *p*-nitrophenyl β-d-lactopyranoside
^conv^MM: Conventional Michaelis–Menten kinetics
^inv^MM: Inverse Michaelis–Menten kinetics
*V*_max_: Maximal rate
*K*_*M*_: Michaelis constant
RAC: Regenerated amorphous cellulose
BMCC: Bacterial microcrystalline cellulose
*Γ*_attack_: Density of attack sites
*Γ*_max_: Maximal adsorption capacity
MD: Molecular dynamics
PAHBAH: *p*-hydroxybenzoic acid hydrazide
PDB: Protein data bank
