# Supplementary material for: Removal of N-linked glycans in cellobiohydrolase Cel7A from Trichoderma reesei reveals higher activity and binding affinity on crystalline cellulose
Source: Biotechnol Biofuels. 2020 Aug 6;13:136. doi: 10.1186/s13068-020-01779-9 (PMC7412794; doi:10.1186/s13068-020-01779-9)
Supplement: Supplementary file 1 — Additional file 1: Fig. S1. Mass distribution profile of full-length TrCel7A from intact protein MS. Fig. S2. DSC scans of all TrCel7A enzymes used in this study. Table S1. Steady-state kinetics and binding affinity parameters (25 °C) of TrCel7A WT and the variants with modified N-glycosylation pattern. Fig. S3. Steady-state kinetic analysis and binding isotherm for TrCel7A enzymes on RAC and BMCC at 50 °C. Fig. S4.InvMM analysis and binding isotherm of TrCel7A CD WT and endoH treated TrCel7A CD on 12 g/L Avicel. Fig. S5. An architecture-based amino acid sequence of the Trichoderma reesei Cel7A. Fig. S6. Phylogenetic tree of the characterized Cel7A sequences from GH7 (CAZy database). Table S2. Statistical comparison by analysis of means of the parameters derived from non-linear fit of Michaelis-Menten and binding isotherms curves (50 °C). Table S3. Statistical comparison by analysis of means of the parameters derived from non-linear fit of Michaelis-Menten and binding isotherms curves (25 °C). [file 13068_2020_1779_MOESM1_ESM.pdf]

Additional File 1

Removal of *N*-linked glycans in cellobiohydrolase Cel7A from *Trichoderma reesei* reveals higher activity and binding affinity on crystalline cellulose

Bartłomiej M. Kołaczkowski[1], Kay S. Schaller[2], Trine Holst Sørensen[3], Günther H. J. Peters[4], Kenneth Jensen[3], Kristian B. R. M. Krogh[3], Peter Westh[2*]

[1] Roskilde University, INM, Universitetsvej 1, Building 28, DK-4000, Roskilde, Denmark

[2]Technical University of Denmark, Department of Biotechnology and Biomedicine. Building 224, DK2800, Kgs. Lyngby, Denmark

[3]Novozymes A/S, Biologiens Vej 2, 2800 Kongens Lyngby, Denmark

[4]Department of Chemistry, Technical University of Denmark, Kemitorvet, DK-2800 Kgs. Lyngby, Denmark

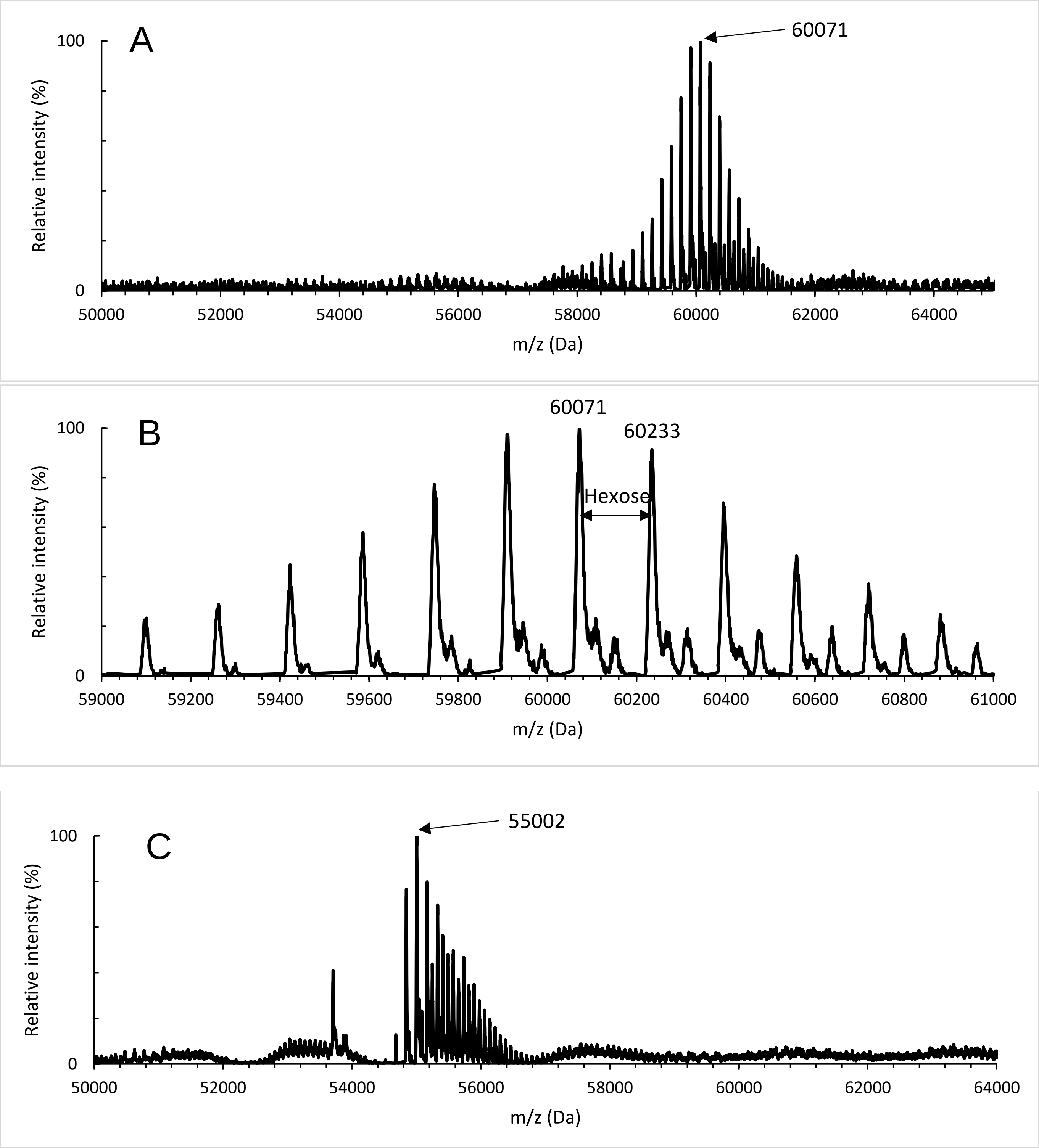
A
60071
Relative intensity (%)
m/z (Da)
B
60071
60233
Hexose
C
55002

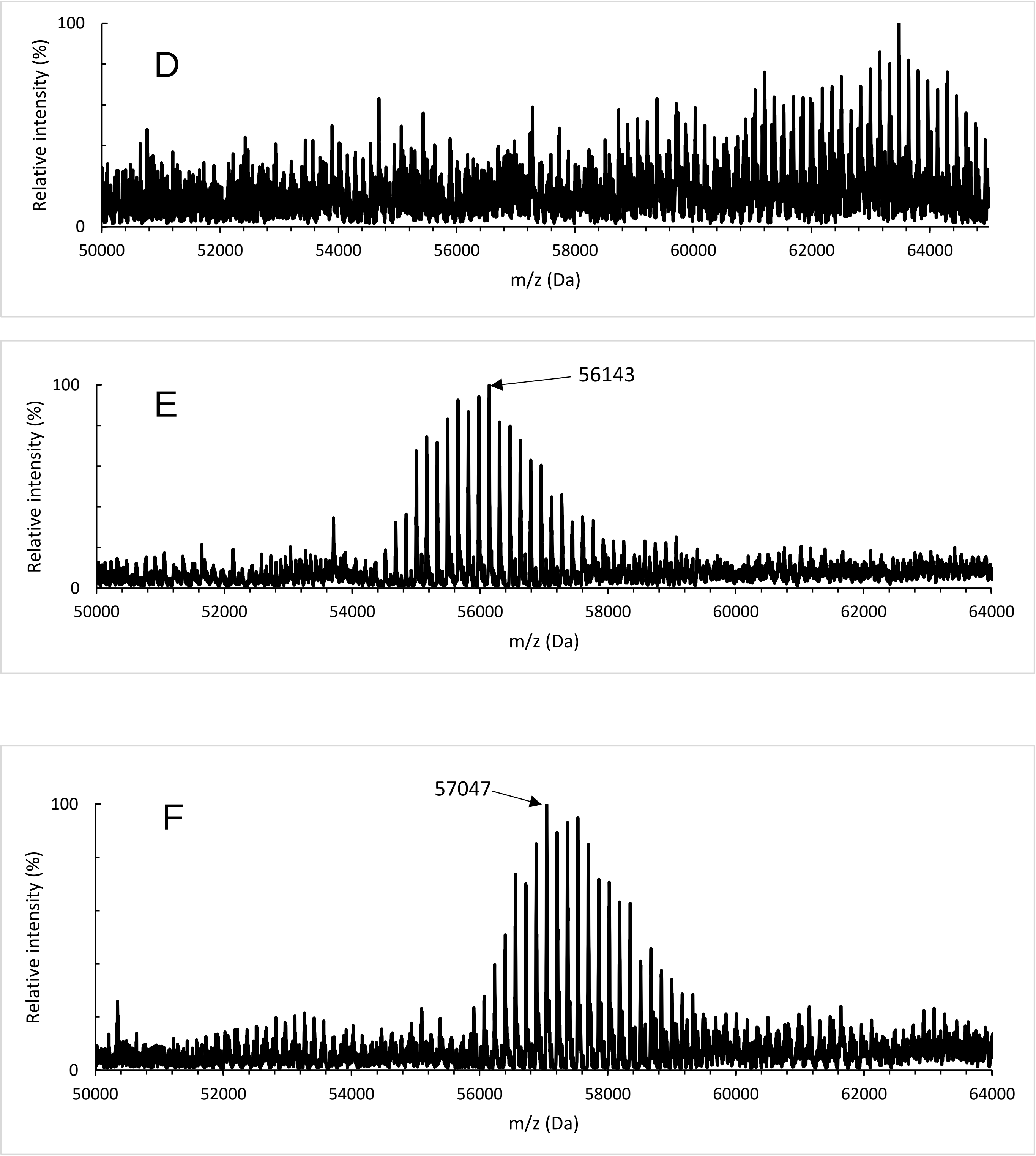
D
Relative intensity (%)
100
0
50000
52000
54000
56000
58000
60000
62000
64000
m/z (Da)
E
56143
Relative intensity (%)
100
0
50000
52000
54000
56000
58000
60000
62000
64000
m/z (Da)
F
57047
Relative intensity (%)
100
0
50000
52000
54000
56000
58000
60000
62000
64000
m/z (Da)

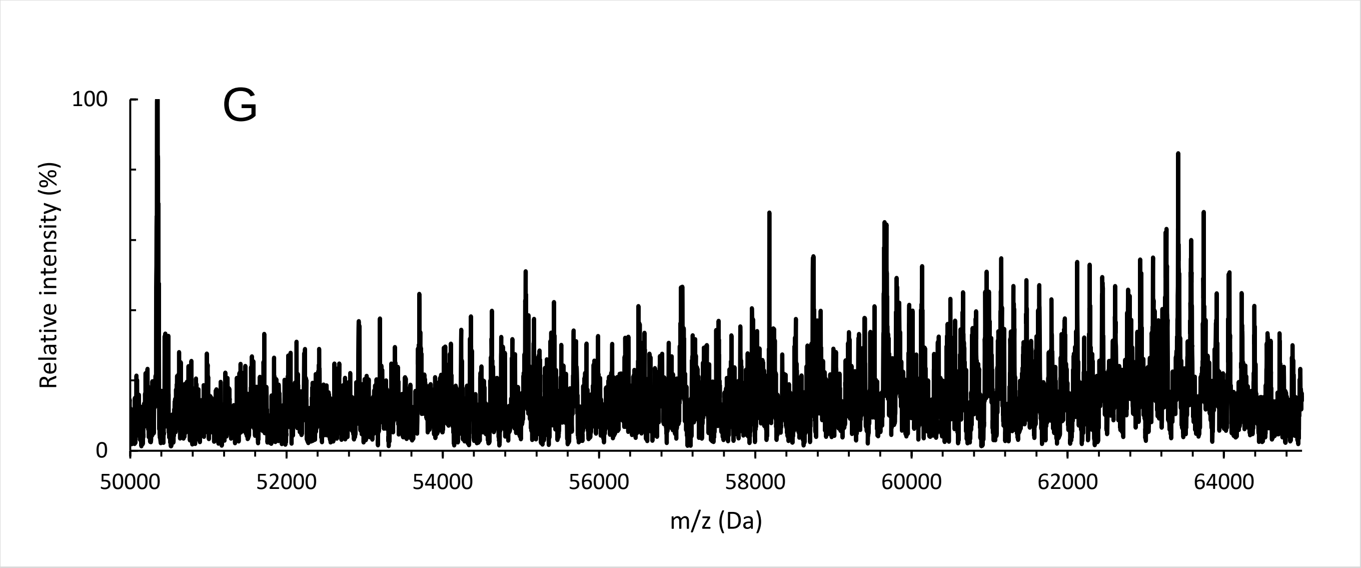

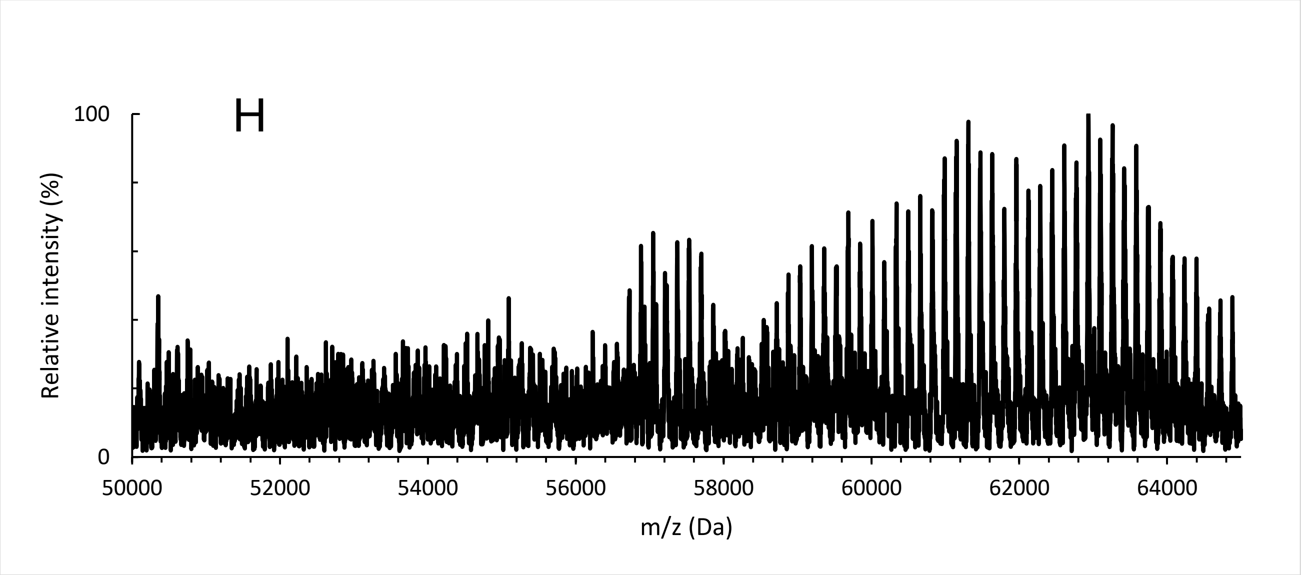

Figure S1. Mass distribution profile of full length TrCel7A enzymes **A)** WT expressed in *T. reesei*, **B)** the zoom of mass spectrum shown in A); the spacing between peaks corresponds to the mass difference of hexose (162 Da) **C)** ΔN-glyc variant expressed in *T. reesei*, **D)** WT expressed in *A. oryzae*, **E)** ΔN-glyc variant expressed in *A. oryzae*, **F)** N45Q variant expressed in *A. oryzae* **G)** N270Q variant expressed in *A. oryzae* and **H)** N384Q variant expressed in *A. oryzae*.

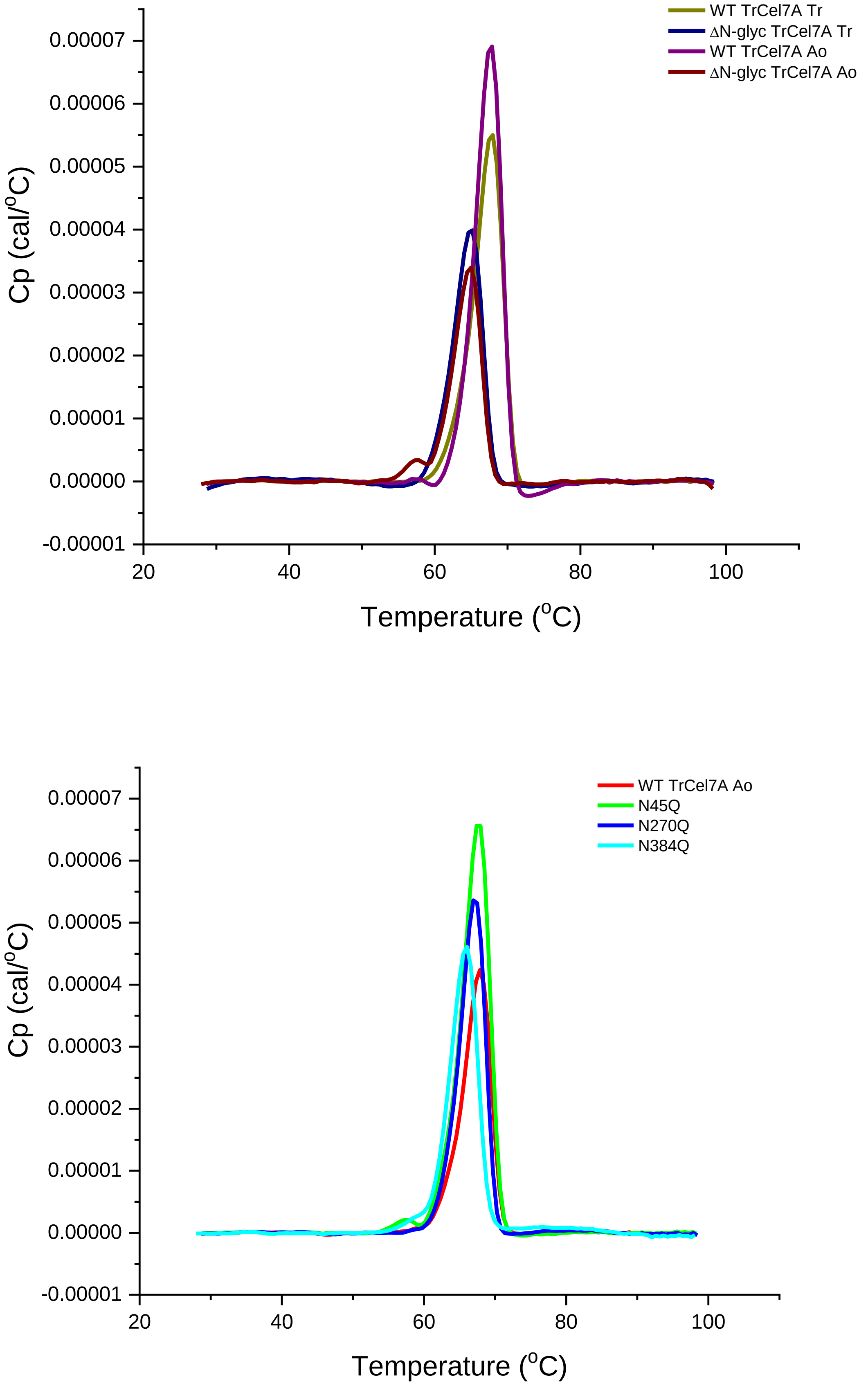

Figure S2. DSC scans of all WT TrCel7A and TrCel7A variants used in this work.

Table S1. Steady-state kinetic and binding affinity parameters (25°C) of *Tr*Cel7A WT and the variants with modified *N*-glycosylation pattern. The parameters were derived from ^{conv}MM, ^{inv}MM and binding isotherm using Avicel, RAC and BMCC. The ± values correspond to the error of non-linear fit of Michaelis-Menten curves and binding isotherm curves. The parameters statistically different from the others at the 0.05 level of significance are indicated with letter 'a' (Table S3).

	^{conv}MM			^{inv}MM		Kinetic substrate accesability	Adsorption isotherms	
Enzyme	${}^{conv}V_{max}/E_0$ (s^{-1})	${}^{conv}K_M$ (g L^{-1})	η ($Lg^{-1}s^{-1}$)	${}^{inv}V_{max}/S_0$ (μmol $g^{-1}s^{-1}$)	${}^{inv}K_M$ (μM)	Γ_{attack} (μmol/g)	Γ_{max} (μmol/g)	K_d (μM)
**Avicel**								
WT_{Ao}	0.14 ± 0.00[a]	4.2 ± 0.5	0.034	0.021 ± 0.001[a]	4.5 ± 0.3[a]	0.15 ± 0.01	0.20 ± 0.01	0.36 ± 0.06
$N45Q_{Ao}$	0.12 ± 0.00	3.7 ± 0.6	0.032	0.028 ± 0.001[a]	5.8 ± 0.3[a]	0.23 ± 0.01	0.29 ± 0.01[a]	0.3 ± 0.03
$N270Q_{Ao}$	0.13 ± 0.00	4.6 ± 0.4	0.027	0.018 ± 0.001[a]	4 ± 0.3[a]	0.14 ± 0.01	0.24 ± 0[a]	0.53 ± 0.04[a]
$N384Q_{Ao}$	0.14 ± 0.00[a]	4.2 ± 0.6	0.034	0.018 ± 0[a]	3.4 ± 0.2	0.13 ± 0.01	0.20 ± 0.01	0.25 ± 0.03
Δ*N*-$glyc_{Ao}$	0.10 ± 0.00[a]	3 ± 0.6	0.032	0.025 ± 0[a]	6.1 ± 0.2[a]	0.25 ± 0.01	0.40 ± 0.01[a]	1 ± 0.07[a]
WT_{Tr}	0.15 ± 0.01[a]	5 ± 0.8	0.029	0.008 ± 0[a]	1.7 ± 0.1[a]	0.05 ± 0.01	0.15 ± 0.01[a]	0.13 ± 0.03[a]
Δ*N*-$glyc_{Tr}$	0.13 ± 0.01	3.3 ± 0.7	0.040	0.012 ± 0[a]	2.8 ± 0.1	0.09 ± 0.01	0.17 ± 0.01[a]	0.16 ± 0.04
**RAC**								
WT_{Ao}	0.11 ± 0.01	0.32 ± 0.11	0.35	0.44 ± 0.01[a]	2.1 ± 0.1	3.9 ± 0.3	3.3 ± 0.1[a]	0.11 ± 0.01
$N45Q_{Ao}$	0.09 ± 0.00	0.14 ± 0.03	0.64	0.43 ± 0.02[a]	2.6 ± 0.3	4.7 ± 0.3	5.6 ± 0.2	0.13 ± 0.02
$N270Q_{Ao}$	0.10 ± 0.00	0.15 ± 0.02	0.66	0.37 ± 0.01	2.2 ± 0.1	3.6 ± 0.1	4.8 ± 0.1	0.2 ± 0.03
$N384Q_{Ao}$	0.10 ± 0.00	0.2 ± 0.04	0.49	0.37 ± 0.01	2.4 ± 0.2	3.8 ± 0.2	4.3 ± 0.2[a]	0.07 ± 0.02
Δ*N*-$glyc_{Ao}$	0.12 ± 0.00[a]	0.29 ± 0.04	0.41	nd	nd	nd	nd	nd
WT_{Tr}	0.11 ± 0.00	0.23 ± 0.04	0.47	0.23 ± 0.01[a]	1.6 ± 0.1[a]	2.1 ± 0.1	6.3 ± 0.3[a]	0.11 ± 0.02
Δ*N*-$glyc_{Tr}$	0.10 ± 0.01	0.25 ± 0.07	0.39	0.32 ± 0.01[a]	2.1 ± 0.2	3.3 ± 0.2	7.1 ± 0.2[a]	0.1 ± 0.02
**BMCC**								
WT_{Ao}	0.35 ± 0.02	0.69 ± 0.09	0.52	0.23 ± 0.01[a]	2.7 ± 0.2[a]	0.64 ± 0.04	3.9 ± 0.2[a]	1.24 ± 0.12[a]
$N45Q_{Ao}$	0.31 ± 0.01	0.53 ± 0.07	0.59	0.30 ± 0.01[a]	2.9 ± 0.2[a]	0.96 ± 0.04	4.0 ± 0.2[a]	0.74 ± 0.09
$N270Q_{Ao}$	0.35 ± 0.02	0.52 ± 0.09	0.67	0.19 ± 0.01	1.1 ± 0.1	0.55 ± 0.04	3.0 ± 0.2	1.27 ± 0.2[a]
$N384Q_{Ao}$	0.38 ± 0.02	0.7 ± 0.13	0.54	0.20 ± 0.01	1.9 ± 0.2[a]	0.52 ± 0.04	4.5 ± 0.3[a]	1.09 ± 0.18[a]
Δ*N*-$glyc_{Ao}$	0.34 ± 0.02	0.43 ± 0.07	0.79	0.28 ± 0.01[a]	1.8 ± 0.2[a]	0.82 ± 0.05	4.4 ± 0.4[a]	1.88 ± 0.42[a]
WT_{Tr}	0.36 ± 0.02	0.49 ± 0.07	0.73	0.14 ± 0[a]	0.6 ± 0.1[a]	0.40 ± 0.02	2.8 ± 0.2	0.47 ± 0.1
Δ*N*-$glyc_{Tr}$	0.38 ± 0.02	0.41 ± 0.07	0.91	0.20 ± 0	0.9 ± 0.1	0.52 ± 0.03	2.4 ± 0.1[a]	0.33 ± 0.05[a]

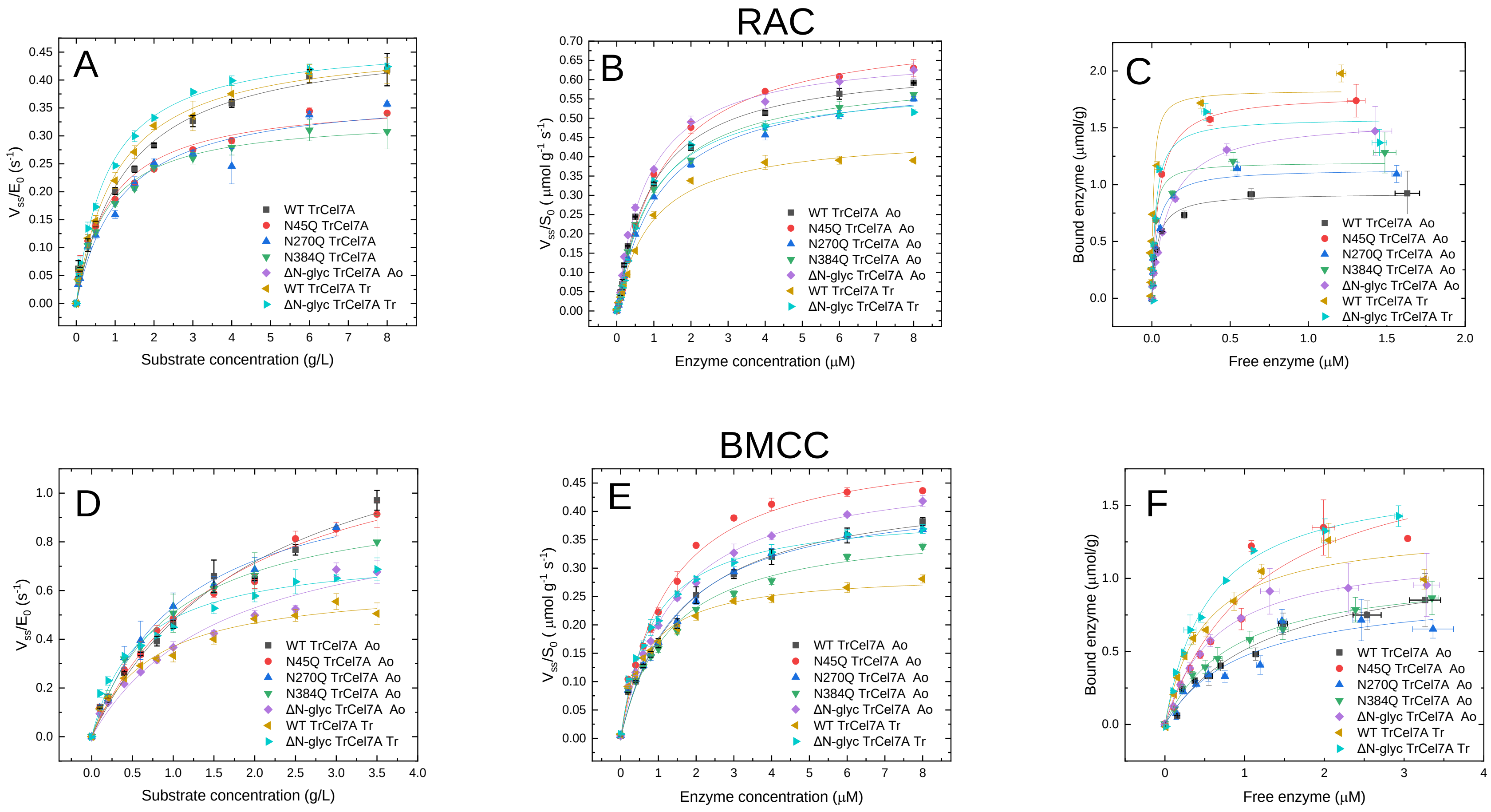

Figure S3. Steady-state kinetic analysis and binding isotherm for *Tr*Cel7A enzymes on RAC and BMCC at 50°C. A) C) In the ^{conv}MM analysis, low enzyme concentration of 50 nM is saturated with increasing RAC concentration (0-8 g/L) and BMCC concentration (0-3.5 g/L), respectively. B) E) In the ^{inv}MM approach, low substrate concentration of 0.4 g/L RAC and 0.75 g/L BMCC, respectively, is saturated with enzyme concentration. The lines indicate a non-linear fit from equation (1) and (2). C) F) Binding isotherm of TrCel7A wt and variants on 0.4 g/L RAC and 0.75 g/L BMCC, respectively, at 50°C. The lines show the fitted Langmuir equation (see equation 4). Error bars represent the standard deviations from a triplicate measurement. Error bars represent standard deviations from triplicate measurements.

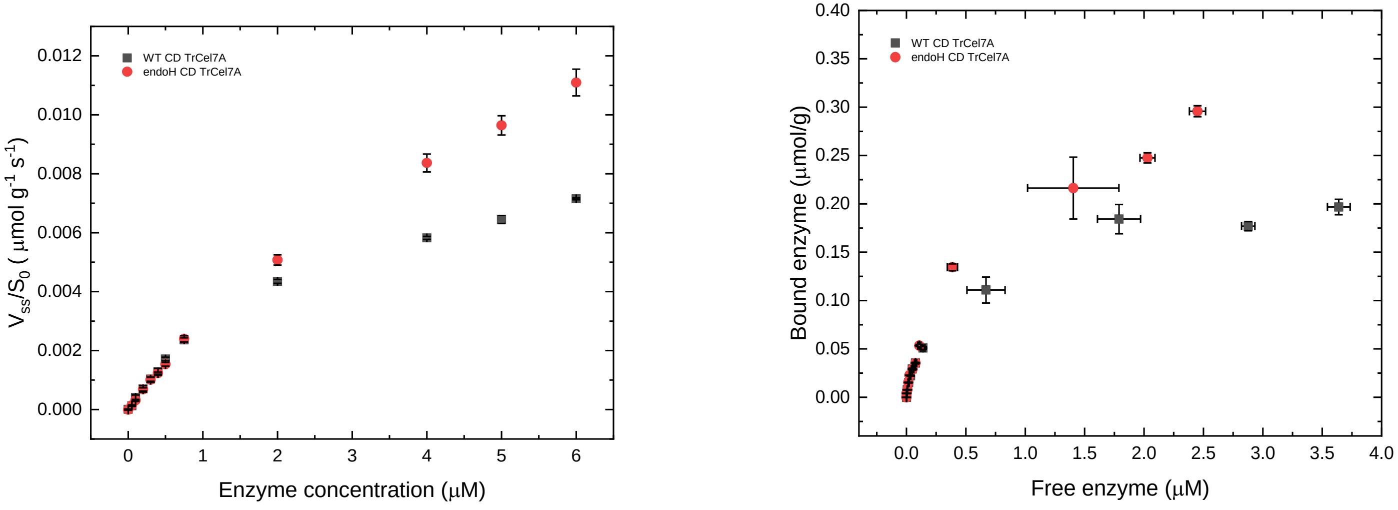

Figure S4. ^{Inv}MM analysis (left) and binding isotherm (right) of TrCel7A CD and endoH treated CD TrCel7A on 12 g/L Avicel. The results show increased activity and binding upon reducing content of *N*-glycosylation. The enzyme was expressed in *A. oryzae*. Error bars represent the standard deviations from triplicate measurements.

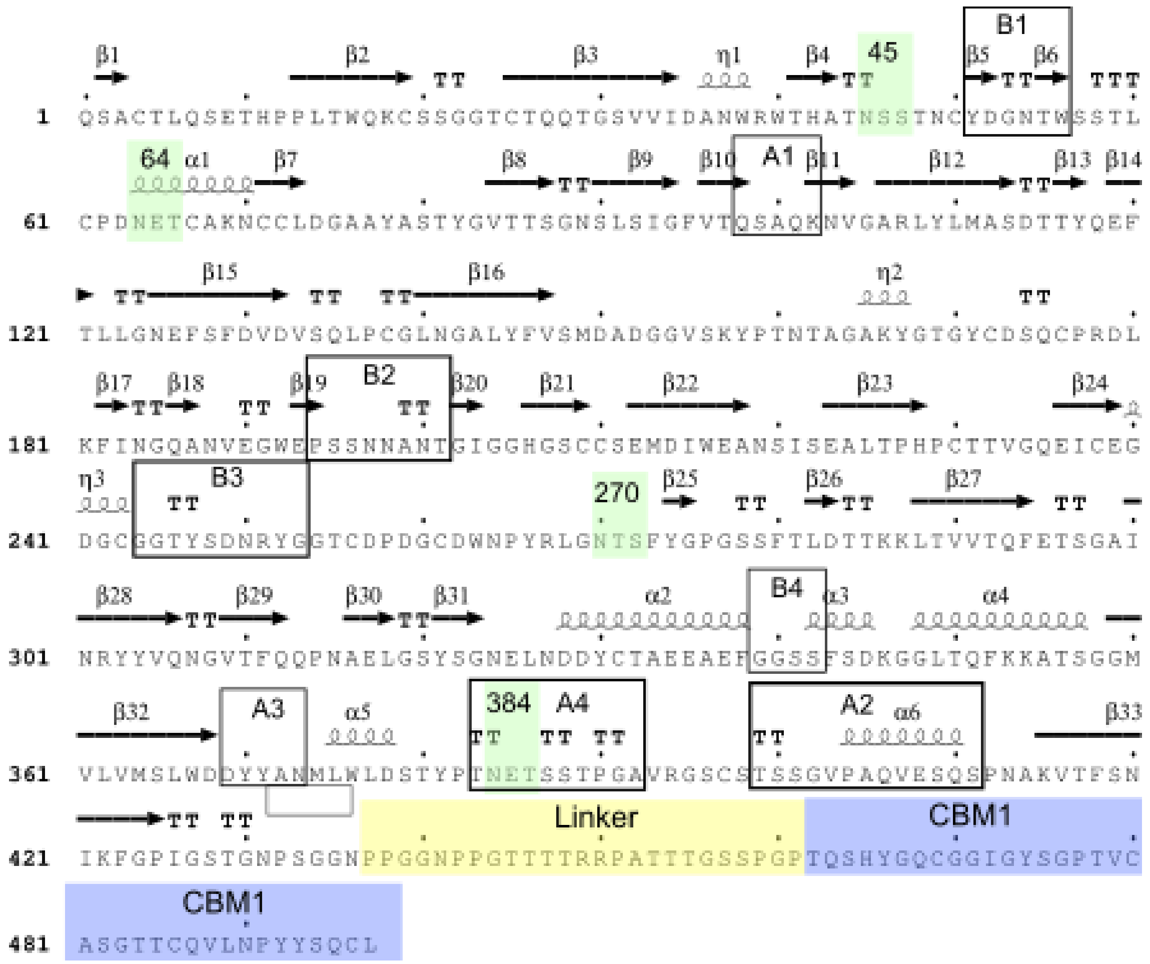

Figure S5. Architecture-based amino acid sequence of the *Trichoderma reesei* Cel7A. Linker and CBM are indicated with yellow and blue color. Experimentally determined secondary structure elements of the catalytic domain are shown on top of the sequence with the following figures and symbols: alpha helices (helices, α and η for a 310-helix), beta strands (arrows, β), turns (T, strict β-turns as TT and strict α-turns as TTT). Green frames indicate all *Tr*Cel7A *N*-glycosylation motifs with the consensus motif (N-X-S/T). Black frames indicate loops characterized by Momeni *et al*. (2013) [1]. The secondary structure annotation were added using ESPript 3.0 web server with default parameters [2].

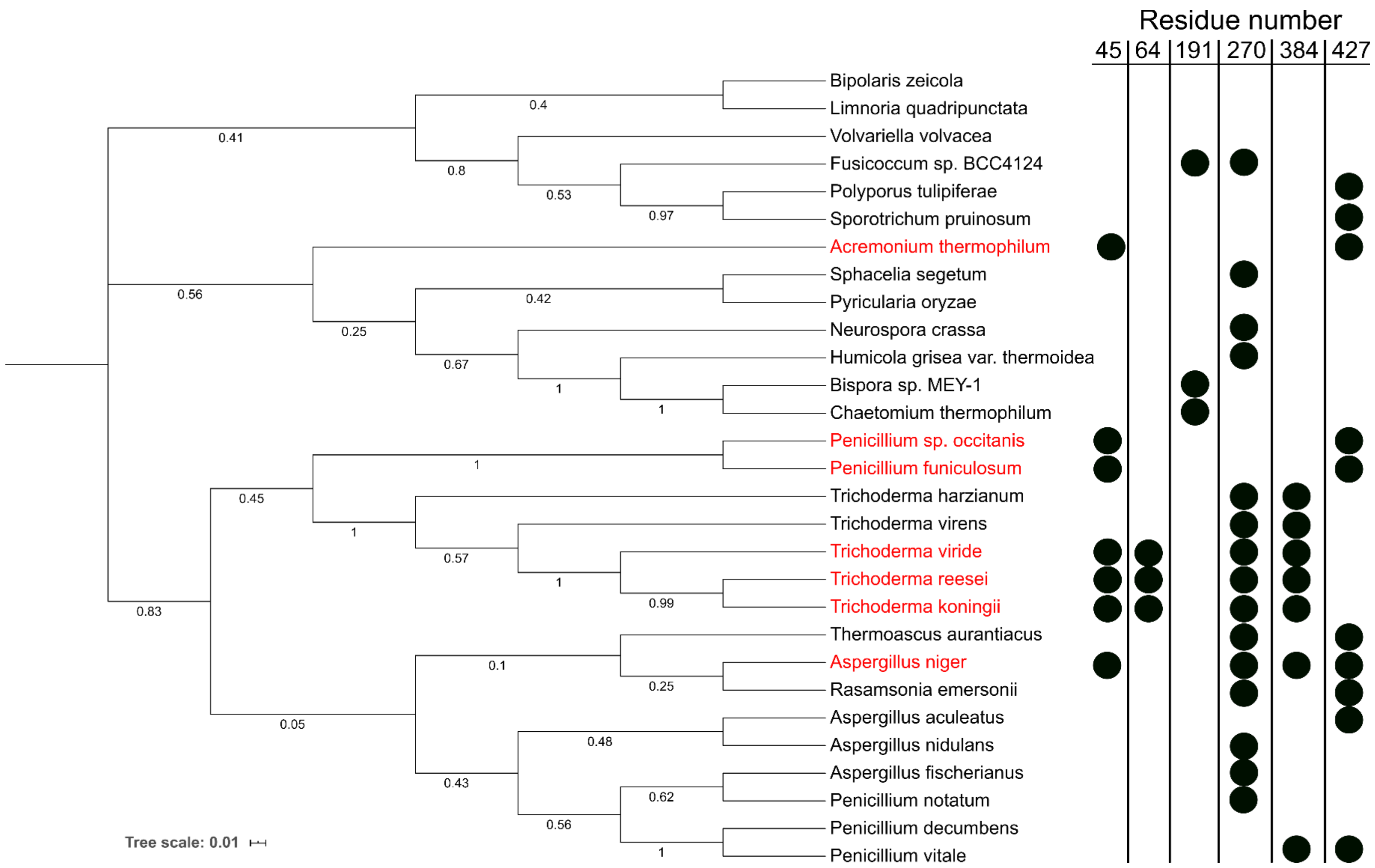

Figure S6. Phylogenetic tree of the characterized Cel7A sequences from GH7. In total 29 sequences were extracted from the CAZy database[3] and used for the multiple sequence alignment , using TrCel7A as the reference sequence. The most common N-glycosylation motifs were annotated in the separate columns and their presence were indicated by black circle. The red colored organisms indicate presence of N-glycosylation site at N45.

Table S2. Statistical comparison by analysis of means of the parameters derived from non-linear fit of Michaelis-Menten and binding isotherms curves (50 °C). The parameters that exceeded upper or lower limit are statistically different from the others at the 0.05 level of significance.

	^{conv}MM								^{inv}MM								Adsorption isotherms							
	$^{conv}V_{max}$				$^{conv}K_M$				$^{inv}V_{max}$				$^{inv}K_M$				Γ_{max}				K_d			
**Avicel**																								
Level	Lower Limit	Estimate	Upper Limit	Limit Exceeded	Lower Limit	Estimate	Upper Limit	Limit Exceeded	Lower Limit	Estimate	Upper Limit	Limit Exceeded	Lower Limit	Estimate	Upper Limit	Limit Exceeded	Lower Limit	Estimate	Upper Limit	Limit Exceeded	Lower Limit	Estimate	Upper Limit	Limit Exceeded
$N45Q_{Ao}$	0.27	0.34	0.36		4.2	11.6	13.7		0.028	0.048	0.034	Upper	1.51	2.92	2.46	Upper	0.08	0.23	0.16	Upper	0.00	0.69	0.81	
$N270Q_{Ao}$	0.27	0.35	0.36		4.2	11.6	13.7		0.029	0.031	0.034		1.51	2.22	2.46		0.08	0.13	0.17		-0.60	1.00	1.41	
$N384Q_{Ao}$	0.27	0.35	0.36		4.8	10.6	13.1		0.028	0.037	0.034	Upper	1.46	2.54	2.52	Upper	0.09	0.10	0.15		0.03	0.30	0.78	
WT_{Ao}	0.25	0.40	0.38	Upper	1.4	18.1	16.5	Upper	0.029	0.034	0.034		1.47	2.45	2.50		0.10	0.10	0.15		0.14	0.26	0.67	
WT_{Tr}	0.28	0.32	0.36		5.3	9.0	12.6		0.030	0.020	0.033	Lower	1.70	1.16	2.28	Lower	0.10	0.08	0.15	Lower	0.08	0.27	0.73	
$\Delta N\text{-glyc}_{Ao}$	0.28	0.24	0.35	Lower	5.9	6.2	12.0		0.027	0.048	0.035	Upper	1.33	3.54	2.64	Upper	0.07	0.27	0.17	Upper	-0.18	1.00	0.99	Upper
$\Delta N\text{-glyc}_{Tr}$	0.28	0.32	0.35		6.1	7.4	11.8		0.029	0.032	0.033		1.67	1.71	2.30		0.09	0.11	0.15		-0.01	0.40	0.82	
**RAC**																								
$N45Q_{Ao}$	0.35	0.37	0.42		0.5	0.9	1.1		0.57	0.74	0.66	Upper	0.73	1.23	1.21	Upper	1.16	1.79	1.61	Upper	0.00	0.05	0.04	Upper
$N270Q_{Ao}$	0.34	0.38	0.43		0.4	1.1	1.3		0.57	0.61	0.66		0.70	1.15	1.23		1.20	1.14	1.58	Lower	0.00	0.03	0.04	
$N384Q_{Ao}$	0.35	0.34	0.42	Lower	0.5	0.8	1.1		0.58	0.62	0.66		0.74	1.03	1.19		1.21	1.20	1.56	Lower	0.01	0.02	0.03	
WT_{Ao}	0.34	0.48	0.43	Upper	0.4	1.3	1.2	Upper	0.58	0.65	0.66		0.77	0.95	1.16		1.21	0.92	1.57	Lower	-0.01	0.03	0.04	
WT_{Tr}	0.34	0.47	0.43	Upper	0.5	1.0	1.1		0.58	0.46	0.66	Lower	0.70	0.94	1.24		1.20	1.83	1.58	Upper	0.01	0.01	0.02	Lower
$\Delta N\text{-glyc}_{Ao}$	0.36	0.30	0.42	Lower	0.6	0.5	1.0	Lower	0.58	0.68	0.65	Upper	0.81	0.82	1.12		1.11	1.58	1.66		-0.04	0.11	0.08	Upper
$\Delta N\text{-glyc}_{Tr}$	0.35	0.47	0.42	Upper	0.6	0.8	1.1		0.58	0.59	0.66		0.76	0.93	1.17		1.19	1.56	1.59		0.01	0.02	0.03	
**BMCC**																								
$N45Q_{Ao}$	0.63	1.33	1.07	Upper	0.3	1.7	1.5	Upper	0.37	0.52	0.44	Upper	0.81	1.23	1.30		0.82	1.96	1.84	Upper	-0.05	1.20	1.26	
$N270Q_{Ao}$	0.65	1.13	1.05	Upper	0.4	1.1	1.3		0.37	0.44	0.45		0.68	1.49	1.43	Upper	0.95	0.93	1.71	Lower	-0.37	0.99	1.59	
$N384Q_{Ao}$	0.68	1.01	1.01		0.5	1.0	1.3		0.37	0.38	0.44		0.70	1.29	1.41		0.99	1.06	1.67		-0.08	0.85	1.30	
WT_{Ao}	0.55	1.46	1.14	Upper	0.1	2.1	1.7	Upper	0.36	0.45	0.45	Upper	0.66	1.59	1.45	Upper	0.85	1.17	1.81		-0.56	1.28	1.77	
WT_{Tr}	0.75	0.63	0.95	Lower	0.5	0.7	1.2		0.38	0.29	0.43	Lower	0.84	0.66	1.27	Lower	1.08	1.34	1.58		0.35	0.48	0.86	
$\Delta N\text{-glyc}_{Ao}$	0.64	0.96	1.05		0.1	1.6	1.6	Upper	0.37	0.48	0.44	Upper	0.75	1.38	1.36	Upper	1.05	1.20	1.61		0.21	0.62	1.01	
$\Delta N\text{-glyc}_{Tr}$	0.76	0.76	0.93		0.7	0.6	1.1	Lower	0.38	0.40	0.43		0.86	0.82	1.25	Lower	1.05	1.71	1.61	Upper	0.36	0.55	0.85	

Table S3. Statistical comparison by analysis of means of the parameters derived from non-linear fit of Michaelis-Menten and binding isotherms curves (25 °C). The parameters that exceeded upper or lower limit are statistically different from the others at the 0.05 level of significance.

	^{conv}MM								^{inv}MM								Adsorption isotherms							
	$^{conv}V_{max}$				$^{conv}K_M$				$^{inv}V_{max}$				$^{inv}K_M$				Γ_{max}				K_d			
**Avicel**																								
Level	Lower Limit	Estimate	Upper Limit	Limit Exceeded	Lower Limit	Estimate	Upper Limit	Limit Exceeded	Lower Limit	Estimate	Upper Limit	Limit Exceeded	Lower Limit	Estimate	Upper Limit	Limit Exceeded	Lower Limit	Estimate	Upper Limit	Limit Exceeded	Lower Limit	Estimate	Upper Limit	Limit Exceeded
$N45Q_{Ao}$	0.12	0.12	0.14		2.2	3.7	5.6		0.012	0.028	0.015	Upper	2.5	5.8	3.9	Upper	0.19	0.29	0.23	Upper	0.12	0.30	0.32	
$N270Q_{Ao}$	0.12	0.13	0.14		1.8	4.6	6.0		0.012	0.018	0.015	Upper	2.6	4.0	3.8	Upper	0.18	0.24	0.23	Upper	0.01	0.53	0.44	Upper
$N384Q_{Ao}$	0.12	0.14	0.14	Upper	2.2	4.2	5.5		0.012	0.018	0.014	Upper	2.8	3.4	3.6		0.19	0.20	0.23		0.12	0.25	0.33	
WT_{Ao}	0.12	0.14	0.14	Upper	2.2	4.2	5.5		0.012	0.021	0.015	Upper	2.6	4.5	3.8	Upper	0.19	0.20	0.23		0.07	0.36	0.38	
WT_{Tr}	0.12	0.15	0.14	Upper	1.9	5.0	5.8		0.013	0.008	0.014	Lower	2.9	1.7	3.6	Lower	0.19	0.15	0.22	Lower	0.15	0.13	0.29	Lower
ΔN-$glyc_{Ao}$	0.12	0.10	0.14	Lower	2.2	3.0	5.6		0.012	0.025	0.015	Upper	2.3	6.1	4.1	Upper	0.17	0.40	0.24	Upper	-0.07	1.00	0.51	Upper
ΔN-$glyc_{Tr}$	0.12	0.13	0.14		2.5	3.3	5.2		0.013	0.012	0.014	Lower	2.8	2.8	3.7		0.19	0.17	0.22	Lower	0.14	0.16	0.30	
**RAC**																								
$N45Q_{Ao}$	0.09	0.09	0.11		0.10	0.16	0.30		0.31	0.43	0.37	Upper	1.7	2.6	2.6		4.6	5.6	5.7		0.04	0.13	0.16	
$N270Q_{Ao}$	0.09	0.10	0.11		0.12	0.15	0.29		0.32	0.37	0.37		1.7	2.2	2.6		4.6	4.8	5.7		-0.01	0.20	0.21	
$N384Q_{Ao}$	0.09	0.10	0.11		0.09	0.20	0.32		0.32	0.37	0.37		1.7	2.3	2.6		4.7	4.3	5.6	Lower	0.06	0.07	0.14	
WT_{Ao}	0.09	0.11	0.12		0.02	0.32	0.38		0.32	0.44	0.37	Upper	1.8	2.1	2.5		4.7	3.3	5.6	Lower	0.02	0.11	0.18	
WT_{Tr}	0.09	0.11	0.11		0.08	0.23	0.33		0.32	0.23	0.37	Lower	1.7	1.6	2.6	Lower	4.6	6.3	5.6	Upper	0.05	0.11	0.15	
ΔN-$glyc_{Ao}$	0.09	0.12	0.12	Upper	0.06	0.29	0.35		nd	nd	nd		nd	nd	nd		nd	nd	nd		nd	nd	nd	
ΔN-$glyc_{Tr}$	0.09	0.10	0.11		0.05	0.25	0.35		0.32	0.32	0.37	Lower	1.7	2.1	2.6		4.6	7.0	5.6	Upper	0.07	0.09	0.13	
**BMCC**																								
$N45Q_{Ao}$	0.31	0.31	0.39		0.24	0.53	0.75		0.16	0.30	0.23	Upper	0.4	2.9	1.8	Upper	2.2	4.0	3.7	Upper	0.18	0.74	0.86	
$N270Q_{Ao}$	0.31	0.35	0.39		0.28	0.52	0.71		0.18	0.19	0.21		0.8	1.1	1.4		2.3	3.0	3.5		-0.14	1.27	1.19	Upper
$N384Q_{Ao}$	0.30	0.38	0.40		0.20	0.70	0.79		0.17	0.20	0.22		0.5	1.9	1.7	Upper	1.9	4.5	3.9	Upper	-0.01	1.09	1.06	Upper
WT_{Ao}	0.30	0.35	0.40		0.19	0.69	0.80		0.16	0.23	0.22	Upper	0.3	2.7	1.9	Upper	2.0	3.9	3.8	Upper	-0.15	1.24	1.19	Upper
WT_{Tr}	0.31	0.36	0.39		0.29	0.49	0.70		0.18	0.14	0.21	Lower	0.9	0.6	1.3	Lower	2.6	2.8	3.3		0.31	0.47	0.73	
ΔN-$glyc_{Ao}$	0.31	0.34	0.39		0.31	0.43	0.67		0.17	0.28	0.21	Upper	0.7	1.8	1.4	Upper	2.1	4.4	3.8	Upper	-0.32	1.88	1.36	Upper
ΔN-$glyc_{Tr}$	0.31	0.38	0.39		0.34	0.41	0.65		0.18	0.20	0.21		0.9	0.9	1.3		2.6	2.4	3.2	Lower	0.37	0.33	0.68	Lower

References

[1] M. H. Momeni *et al.*, "Structural, biochemical, and computational characterization of the glycoside hydrolase family 7 cellobiohydrolase of the tree-killing fungus heterobasidion irregulare," *J. Biol. Chem.*, vol. 288, no. 8, pp. 5861–5872, 2013.

[2] X. Robert and P. Gouet, "Deciphering key features in protein structures with the new ENDscript server," *Nucleic Acids Res.*, vol. 42, no. W1, pp. 320–324, 2014.

[3] V. Lombard, H. Golaconda Ramulu, E. Drula, P. M. Coutinho, and B. Henrissat, "The carbohydrate-active enzymes database (CAZy) in 2013," *Nucleic Acids Res.*, vol. 42, no. D1, pp. 490–495, 2014.
